# Body shape phenotypes of multiple anthropometric traits and cancer risk: a multi-national cohort study

**DOI:** 10.1038/s41416-022-02071-3

**Published:** 2022-12-02

**Authors:** Anja M. Sedlmeier, Vivian Viallon, Pietro Ferrari, Laia Peruchet-Noray, Emma Fontvieille, Amina Amadou, Nazlisadat Seyed Khoei, Andrea Weber, Hansjörg Baurecht, Alicia K. Heath, Kostas Tsilidis, Rudolf Kaaks, Verena Katzke, Elif Inan-Eroglu, Matthias B. Schulze, Kim Overvad, Catalina Bonet, Esther Ubago-Guisado, María-Dolores Chirlaque, Eva Ardanaz, Aurora Perez-Cornago, Valeria Pala, Rosario Tumino, Carlotta Sacerdote, Fabrizio Pasanisi, Kristin B. Borch, Charlotta Rylander, Elisabete Weiderpass, Marc J. Gunter, Béatrice Fervers, Michael F. Leitzmann, Heinz Freisling

**Affiliations:** 1 Department of Epidemiology and Preventive Medicine, University of Regensburg, Regensburg, Germany; 2 International Agency for Research on Cancer (IARC), Nutrition and Metabolism Branch, Lyon, France; 3 Department of Clinical Sciences, Faculty of Medicine, University of Barcelona, Barcelona, Spain; 4 Department of Prevention Cancer Environment, Centre Léon Bérard, Lyon, France; 5 INSERM UMR1296 Radiation: Defense, Health, Environment, Lyon, France; 6 Department of Nutritional Sciences, Faculty of Life Sciences, University of Vienna, Vienna, Austria; 7 Department of Epidemiology and Biostatistics, School of Public Health, Imperial College London, London, UK; 8 Department of Hygiene and Epidemiology, University of Ioannina School of Medicine, Ioannina, Greece; 9 Division of Cancer Epidemiology, German Cancer Research Center (DKFZ), Heidelberg, Germany; 10 Department of Molecular Epidemiology, German Institute of Human Nutrition Potsdam-Rehbruecke, Nuthetal, Germany; 11 Institute of Nutritional Science, University of Potsdam, Nuthetal, Germany; 12 Department of Public Health, Aarhus University, Aarhus, Denmark; 13 Unit of Nutrition and Cancer, Catalan Institute of Oncology (ICO), L’Hospitalet de Llobregat, Barcelona, Spain; 14 Nutrition and Cancer Group, Epidemiology, Public Health, Cancer Prevention and Palliative Care Program, Bellvitge Biomedical Research Institute - IDIBELL, L’Hospitalet de Llobregat, Barcelona, Spain; 15 Escuela Andaluza de Salud Pública (EASP), Granada, Spain; 16 Instituto de Investigación Biosanitaria ibs.GRANADA, Granada, Spain; 17 CIBER Epidemiology and Public Health CIBERESP, Madrid, Spain; 18 Department of Epidemiology, Regional Health Council, Murcia, Spain; 19 IMIB-Arrixaca, Murcia University, Murcia, Spain; 20 Navarra Public Health Institute, IdiSNA, Pamplona, Spain; 21 Cancer Epidemiology Unit, Nuffield Department of Population Health, University of Oxford, Oxford, UK; 22 Epidemiology and Prevention Unit, Fondazione IRCCS Istituto Nazionale dei Tumori, Milano, Italy; 23 Hyblean Association for Epidemiological Research, AIRE - ONLUS, Ragusa, Italy; 24 Unit of Cancer Epidemiology, Città della Salute e della Scienza University-Hospital, Turin, Italy; 25 Clinical Nutrition Unit, Department of Clinical Medicine and Surgery, Federico II University Hospital, Naples, Italy; 26 Department of Community Medicine, Faculty of Health Sciences, UiT The Arctic University of Norway, Tromsø, Norway

**Keywords:** Risk factors, Cancer epidemiology

## Abstract

**Background:**

Classical anthropometric traits may fail to fully represent the relationship of weight, adiposity, and height with cancer risk. We investigated the associations of body shape phenotypes with the risk of overall and site-specific cancers.

**Methods:**

We derived four distinct body shape phenotypes from principal component (PC) analysis on height, weight, body mass index (BMI), waist (WC) and hip circumferences (HC), and waist-to-hip ratio (WHR). The study included 340,152 men and women from 9 European countries, aged mostly 35–65 years at recruitment (1990–2000) in the European Prospective Investigation into Cancer and Nutrition (EPIC) study. Cox proportional hazards regression was used to estimate multivariable-adjusted hazard ratios (HRs) and 95% confidence intervals (CIs).

**Results:**

After a median follow-up of 15.3 years, 47,110 incident cancer cases were recorded. PC1 (overall adiposity) was positively associated with the risk of overall cancer, with a HR per 1 standard deviation (SD) increment equal to 1.07 (95% confidence interval 1.05 to 1.08). Positive associations were observed with 10 cancer types, with HRs (per 1 SD) ranging from 1.36 (1.30–1.42) for endometrial cancer to 1.08 (1.03–1.13) for rectal cancer. PC2 (tall stature with low WHR) was positively associated with the risk of overall cancer (1.03; 1.02–1.04) and five cancer types which were not associated with PC1. PC3 (tall stature with high WHR) was positively associated with the risk of overall cancer (1.04; 1.03–1.05) and 12 cancer types. PC4 (high BMI and weight with low WC and HC) was not associated with overall risk of cancer (1.00; 0.99–1.01).

**Conclusions:**

In this multi-national study, distinct body shape phenotypes were positively associated with the incidence of 17 different cancers and overall cancer.

## Introduction

General adiposity, usually defined by a high body mass index (BMI), is an established risk factor for several malignancies, including cancers of the oesophagus (adenocarcinoma), pancreas, colon, rectum, breast (postmenopausal), corpus uteri, kidney, gallbladder, stomach (cardia), liver, ovary, prostate (advanced stage), mouth, pharynx, multiple myeloma, and meningioma [1, 2]. In regions with high obesity prevalence, 4–9% of the cancer burden is attributable to a BMI ≥ 25 kg/m^2^ [3].

However, the obesity-attributable cancer burden is likely underestimated [4], because BMI neither differentiates between muscle and fat mass nor does it capture body fat distribution [3]. Waist or hip circumferences (WC or HC) and waist-to-hip ratio (WHR) represent surrogate markers of body fat distribution and have shown associations with cancer similar to those seen with BMI [5, 6]. Likely explanations are that these indicators track with general adiposity and are also highly inter-correlated [3], rendering them relatively non-specific regarding cancer risk. Adult attained height is an established risk factor for at least eight different types of cancer including pancreas, colorectum, endometrium, ovary, prostate, kidney, skin, and breast (pre- and postmenopausal) [7–12]. Although increased cancer risk due to height is largely independent of obesity and could be explained by an increased cell number in taller individuals [13], some overlap with obesity due to shared mechanistic pathways (e.g., elevated insulin-like growth factor 1 [IGF-1] levels) [14] cannot entirely be excluded. Taken together, classical anthropometric traits may fail to fully represent the complex relations of relative weight, adiposity, and height to cancer risk [15].

In a meta-analysis of 65 studies, Ried et al. [16] combined six anthropometric traits (i.e. weight, height, BMI, WC, HC, and WHR) using principal component analysis (PCA) and derived four principal components (PCs) for body shape phenotypes, which together explained over 99% of the total variation in these anthropometric traits [16]. The PCs showed large agreement across studies and between men and women [16]. The findings of Ried et al. suggest that the body shape phenotypes represent information that is not fully captured by individual anthropometric traits [16]. The body shape phenotypes showed differential associations with various indicators of metabolic health, such as elevated blood lipids, blood glucose, and insulin sensitivity [16], which are candidate mediators underlying the association between obesity and carcinogenesis [17]. Whether different body shapes are associated with cancer risk is unknown.

We used data of the European Prospective Investigation into Cancer and Nutrition (EPIC) study, applied the approach of Ried et al. [16] to derive four distinct body shape phenotypes and investigated associations of these body shape phenotypes with overall and site-specific cancer risk.

## Materials and methods

### Study population

The EPIC study is a prospective multicenter cohort investigating the association between lifestyle factors and cancer and other chronic diseases [18]. Between 1992 and 2000, approximately 520,000 men and women mostly aged between 35 and 65 years from 22 study centres in 9 different European countries (Denmark, France, Germany, Italy, the Netherlands, Norway, Spain, Sweden, and the United Kingdom) were recruited. Participants were selected from the general population, with few exceptions: in France, female employees in state schools were recruited; in Utrecht (Netherlands) and Florence (Italy), women who had participated in breast cancer screening were included; and in some centres in Spain and Italy, registered members from blood donor registries were selected. The Oxford (UK) cohort recruited half of the participants from groups of vegetarians and vegans [18–20]. Data from Greece were unavailable for this analysis.

All participants provided written informed consent, and approval for the study was obtained from the International Agency for Research on Cancer (IARC) ethics review panel (No. 20-34) and from all recruiting institutions. At recruitment, information on socioeconomic and lifestyle factors and medical history were obtained using questionnaires.

After exclusions, the current analysis comprised 340,152 participants (118,218 men and 221,934 women: Supplementary Fig. 1).

### Assessment of anthropometric measures

Anthropometric measurements followed standard protocols, except in France and Oxford (UK), where data on body weight were based on self-report [21]. The accuracy of self-reported anthropometric measures was improved by using prediction equations derived from participants with both measured and self-reported data at baseline. These recalibrated self-reported anthropometric measures are valid for identifying associations in epidemiologic studies [22].

Body weight was measured without shoes to the nearest 0.1 kg and height to the nearest 0.1 cm or 0.5 cm. BMI was calculated as body weight (kilograms, kg) divided by height in metres squared (m^2^). WC was measured at the narrowest circumference of the torso or midway between the lowest ribcage and the highest point of the iliac crest. HC was determined horizontally at the level of the greatest lateral extent of the hips or above the buttocks. Body circumferences were rounded to the nearest centimetre. WHR was calculated as WC (cm) divided by HC (cm). To reduce heterogeneity due to protocol differences between centres, body weight, WC and HC of each participant were corrected for clothing worn during measurement [22]. Furthermore, centre-, age-, and sex-specific mean values for weight, height, WC, and HC were imputed for individuals with neither self-reported nor measured anthropometric data.

### Ascertainment of cancer cases

Cancer cases were mainly identified through population-based cancer registries. In Germany and France, cancers were identified using health insurance records, cancer and pathology registries, and active follow-up of participants and next of kin [18]. Complete follow-up occurred between December 2009 and December 2013, depending on the centre.

Incident cancer cases were coded using the International Classification of Diseases and the third revision of the International Classification of Diseases for Oncology (malignant primary site) [23]. Detailed information on tumour topography is provided in Supplementary Table 1. The present analyses focused on the first primary cancer diagnosis. Participants who later developed a subsequent cancer were considered a case at the time of their first cancer. Endpoints were defined as all cancers combined and individual cancer types (bladder, brain and central nervous system (CNS), breast (postmenopausal and premenopausal), cervix, colon, corpus uteri, oesophagus (adeno and squamous cell carcinomas (SCC)), gallbladder, kidney, larynx, lips, oral cavity and pharynx, liver, lung, malignant melanoma, myeloma, ovary, pancreas, prostate, rectum, stomach (cardia and non-cardia), and thyroid). Cancer types with fewer than 100 cases were not considered for analyses.

### Statistical analysis

We performed PCA on the standardised residuals of height, weight, BMI, WC, HC, and WHR. Residuals were computed in separate linear regression models of the six anthropometric traits on age, sex, and study centre. The age, sex, and centre adjustment should facilitate comparability across study populations by removing the extraneous variability introduced by these variables. This resulted in a set of six PCs representing orthogonal linear combinations of the six traits [24]; i.e., each component represented a weighted sum of the six transformed traits and was independent of the other components. For better characterisation of each body shape, the mean values of each trait among participants in the top and bottom 5% proportions are presented alongside their visualisations using https://bodyvisualizer.com/. Pearson’s correlation coefficients between all anthropometric measures and the PCs were also calculated. To minimise possible influence of outliers, PC data were winsorized at 1 and 99% [25].

We used Cox proportional hazards models with age as the underlying time metric to estimate hazard ratios (HRs) and 95% confidence intervals (CIs). Age at recruitment was the entry time, and age at the first primary cancer diagnosis, age at end of follow-up, age at loss-to-follow-up, or age at time of death, whichever came first, were the exit time. HRs and 95% CIs per 1 standard deviation (SD) increment of each PC were calculated to allow body shapes to be compared. Models were stratified by age at recruitment (in 5-year groups), sex, and study centre. To avoid a duplication of tests for this novel exposure across 24 types of cancers and because the loadings for the anthropometric traits across the four PCs were very similar among men and women, we provided results for men and women combined.

We fitted a crude model that included the four body shape PCs. Potential confounding variables for the multivariable models were identified a priori using Directed Acyclic Graphs (Supplementary Fig. 2) [26, 27]. Model selection was based on Akaike’s Information Criterion. Proportional hazards assumptions were tested using scaled Schoenfeld residuals [28]. Departure from linearity for all continuous exposure variables was assessed by log-likelihood ratio tests and if necessary, restricted cubic splines were used with three knots placed at the 10th, 50th, and 90th percentiles [24].

Analyses were repeated among never and current smokers to assess potential residual confounding by smoking and were stratified by median age (52.3 years) to address potential changes of anthropometric measures. In addition, to control for potential reverse causation, sensitivity analyses were performed excluding the first 2 years of follow-up.

All statistical tests were two-sided and Bonferroni-corrected *P* values ≤0.001 (~0.05/96 tests) were considered statistically significant. Analyses were performed using R version 4.0.3 (R Foundation for Statistical Computing, Vienna, Austria, 2020).

## Results

**Fig. 1:**
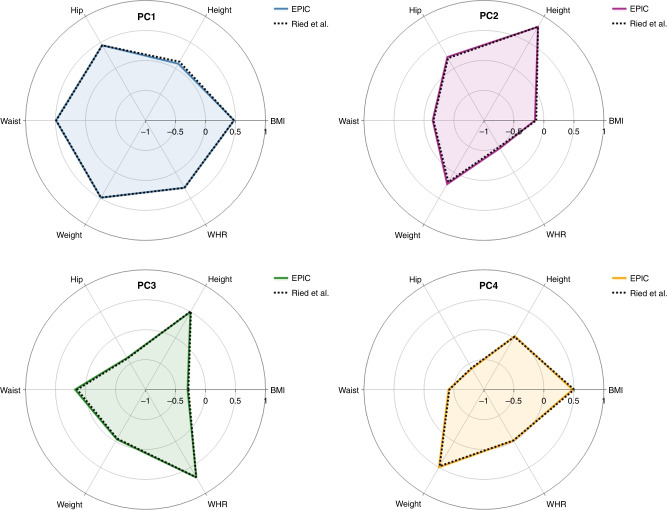
Loadings for the four different body shape phenotypes. PC1: blue; PC2: magenta; PC3: green; PC4: orange.

The first four PCs together explained 99.8% of the total variation of the six anthropometric variables. Thus, all analyses were restricted to these PCs (Table 1). Each PC described a distinct body morphology (Fig. 1). The loadings for each anthropometric trait are presented in Table 1 (men and women combined), which were very similar among men and women (Supplementary Tables 2 and 3). For better comparability with Ried et al. [16], the directionality of PC4 was reversed.

**Table 1:** Loadings and explained variance of the principal components (PCs) for the analytic study population in EPIC (*n* = 340,152) and average loadings and average explained variance derived by Ried et al. [16] labelled “avPC”.

	PC1	avPC1^a^	PC2	avPC2	PC3	avPC3	PC4^a^	avPC4^a^	PC5	avPC5	PC6	avPC6
Height	0.092	0.131	0.801	0.803	0.497	0.513	0.029	0.018	0.262	0.265	−0.183	−0.062
Weight	0.487	0.486	0.224	0.186	−0.042	−0.056	0.497	0.475	−0.557	−0.690	0.394	0.155
BMI	0.476	0.473	−0.149	−0.128	−0.299	−0.284	0.479	0.504	0.539	0.635	−0.377	−0.148
Waist circumference	0.493	0.488	−0.146	−0.159	0.175	0.138	−0.411	−0.424	−0.428	−0.163	−0.594	−0.715
Hip circumference	0.446	0.444	0.219	0.197	−0.389	−0.399	−0.595	−0.583	0.272	0.110	0.417	0.503
WHR	0.295	0.297	−0.466	−0.490	0.693	0.689	−0.015	−0.019	0.272	0.105	0.377	0.432
Explained variance [%]	63.04	64.37	19.60	18.46	14.39	13.79	2.81	2.97	0.11	0.26	0.06	0.15

*avPC* average principal component, *BMI* body mass index, *PC* principal component, *WHR* waist-to-hip ratio.

^a^Loadings for avPC1, PC4 and avPC4 were inverted.

PC1 explained 63.0% of the total variation, with high loadings for all anthropometric measures except height, describing individuals characterised by general obesity (Supplementary Fig. 3). PC2 (19.6% of total variation) was characterised by opposite loadings for height and WHR (Supplementary Fig. 4), mainly discriminating between tall individuals with low WHR and short individuals with high WHR. PC3 (14.4% of the total variation) was characterised by loadings for height and WHR in the same direction, HC loadings in the opposite direction, and low loadings for BMI (Supplementary Fig. 5), distinguishing between tall individuals with high WHR but low HC and short individuals with low WHR and high HC. PC4 represents a rare phenotype explaining only 2.8% of the total variation and was characterised by high loadings for body weight and BMI and low loadings for WC and HC (Supplementary Fig. 6). Pearson’s correlation coefficients for the six anthropometric variables were consistent with the loadings for the individual PCs (Supplementary Fig. 7).

### Baseline characteristics

Baseline characteristics are presented for sex-specific quintiles of loadings for PC1 (Table 2). Anthropometric measures were notably larger in quintile 5 than in quintile 1, except for WHR and height. Participants in the lowest two PC1 quintiles had a healthier diet, a higher educational attainment, were more physically active, and smoked more frequently compared with the top 20% of the study population. Additional baseline characteristics are provided in Supplementary Table 4.

**Table 2:** Characteristics of participants according to sex-specific quintiles of loadings of principal component 1 (overall adiposity) in EPIC^a^.

	Men (*n* = 118,218)					Women (*n* = 221,934)				
	Q1	Q2	Q3	Q4	Q5	Q1	Q2	Q3	Q4	Q5
	Mean (SD) or %					Mean (SD) or %				
No. (%)	23,764 (20.1)	23,393 (19.8)	23,971 (20.3)	23,570 (19.9)	23,520 (19.9)	44,781 (20.2)	44,356 (20.0)	43,827 (19.7)	44,628 (20.1)	44,342 (20.0)
Age at recruitment (years)	53.8 (10.0)	52.9 (9.8)	52.3 (9.7)	52.2 (9.5)	52.6 (9.0)	51.5 (10.3)	50.0 (10.7)	49.9 (11.0)	50.9 (10.7)	52.1 (9.7)
Median follow-up time (years)	15.2	15.5	15.5	15.5	15.1	15.3	15.3	15.4	15.3	15.2
Anthropometric variables										
Weight (kg)	67.3 (5.7)	74.7 (4.6)	79.7 (4.6)	85.5 (4.8)	97.6 (9.6)	54.1 (4.8)	59.8 (4.5)	64.3 (4.6)	69.9 (5.1)	83.1 (10.5)
Height (kg)	172.0 (7.0)	173.8 (6.9)	175.0 (6.8)	176.0 (7.0)	177.3 (7.2)	160.2 (6.2)	161.9 (6.4)	162.6 (6.6)	162.8 (6.9)	162.6 (6.9)
Body mass index (kg/m^2^)	22.8 (2.0)	24.8 (1.9)	26.1 (1.9)	27.7 (2.0)	31.1 (3.2)	21.1 (1.9)	22.9 (2.0)	24.4 (2.2)	26.5 (2.4)	31.5 (4.2)
Waist circumference (cm)	83.2 (5.8)	89.6 (4.9)	93.5 (5.0)	98.0 (5.3)	107.5 (8.1)	68.9 (4.8)	73.6 (5.2)	77.6 (5.8)	83.1 (6.4)	95.0 (9.7)
Hip circumference (cm)	93.9 (4.4)	97.9 (3.8)	100.2 (3.9)	102.8 (4.2)	108.5 (6.6)	92.1 (4.8)	96.2 (4.6)	99.3 (4.8)	103.1 (5.3)	112.4 (8.8)
Waist-to-hip ratio	0.9 (0.1)	0.9 (0.1)	0.9 (0.1)	1.0 (0.1)	1.0 (0.1)	0.7 (0.0)	0.8 (0.1)	0.8 (0.1)	0.8 (0.1)	0.8 (0.1)
Mediterranean Diet Score (%)^b^										
Low	35.1	32.5	32.5	34.8	40.6	24.1	21.4	21.4	23.3	28.2
Medium	41.9	43.2	42.7	42.2	41.4	45.8	45.4	45.0	45.3	45.2
High	23.0	24.3	24.8	23.0	18.1	30.2	33.2	33.6	31.4	26.6
Highest school level (%)										
None	3.6	3.7	3.8	3.7	3.3	2.8	3.4	4.2	5.7	7.7
Primary school completed	27.2	26.6	27.9	30.1	32.9	23.5	22.2	23.5	27.3	32.9
Technical/professional school	24.6	25.1	24.5	25.3	26.8	27.0	25.3	24.8	24.6	25.5
Secondary school	12.3	12.1	12.0	11.8	10.9	18.5	18.8	18.7	17.6	14.7
Longer education (incl. University degree)	30.3	30.5	29.6	26.8	23.7	25.2	26.5	24.8	20.8	15.6
Not specified	2.1	2.1	2.2	2.3	2.3	2.9	3.8	3.9	4.0	3.7
Physical activity (%)^c^										
Inactive	16.0	15.8	16.0	17.6	21.4	21.3	20.0	21.0	24.1	29.6
Moderately inactive	29.3	30.6	31.6	31.6	31.6	35.1	35.8	36.0	35.4	34.2
Moderately active	24.1	24.7	24.0	24.0	22.3	23.0	24.0	23.5	22.0	19.5
Active	28.1	26.9	26.5	25.1	23.0	19.3	18.8	18.2	17.2	15.4
Unknown	2.5	2.1	1.9	1.8	1.7	1.3	1.4	1.3	1.3	1.3
Smoking status (%)										
Never	33.9	33.2	32.6	30.0	27.4	51.7	55.5	56.3	56.9	56.8
Former	30.9	36.7	38.7	41.4	43.9	22.4	23.7	24.1	24.4	24.8
Current	34.8	29.6	28.3	28.1	28.3	25.4	20.1	18.9	18.1	17.8
Unknown	0.4	0.5	0.4	0.5	0.4	0.5	0.7	0.7	0.7	0.6
Alcohol intake (g/day)	20 (22)	21 (22)	22 (23)	23 (23)	24 (27)	9 (12)	9 (12)	9 (12)	9 (12)	8 (12)

*EPIC* European Investigation into Cancer and Nutrition.

^a^Sex-specific quintiles of loadings of principal component 1 were defined by their distribution (20th quantile, 40th quantile, 60th quantile, 80th quantile); for men: 20th quantile = −0.73, 40th quantile = −0.27, 60th quantile = 0.16, 80th quantile = 0.71; for women: 20th quantile = −0.82, 40th quantile = −0.37, 60th quantile = 0.07, 80th quantile = 0.72.

^b^Adherence to the Mediterranean dietary pattern, based on nine dietary components: intake of fruits and nuts, vegetables, legumes, cereals, lipids, fish, dairy and meat products, and alcohol [39].

^c^Physical activity is defined according to the Cambridge physical activity index [40].

### Body shapes and cancer risk

After a median follow-up of 15.3 years (interquartile range = 12.8–16.8 years) and 4,841,860 person-years, 47,110 incident cancer cases were diagnosed. Among participants, 65% were women; the mean age at recruitment was 50.9 years (SD = ±10.5 years) for women and 52.7 years (SD = ±9.6 years) for men.

### Results for PC1 (overall adiposity)

**Fig. 2:**
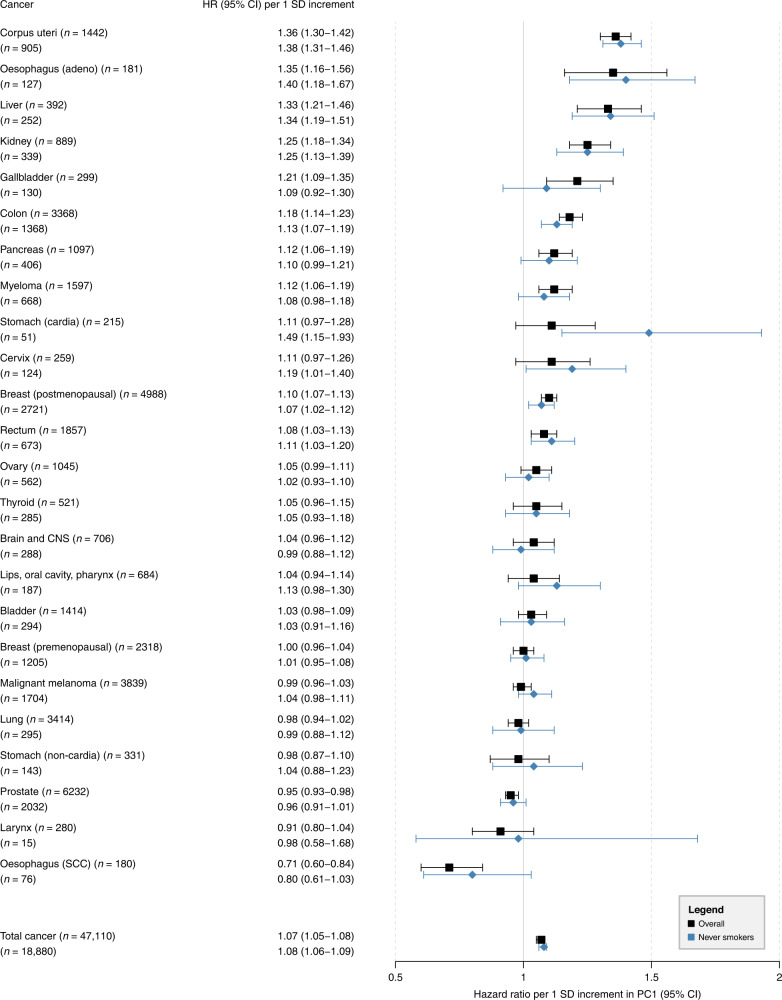
Hazard ratios (HRs) for total cancer and 24 cancer subtypes per 1 SD increment in the first principal component (PC1; overall adiposity). HRs with corresponding 95% confidence intervals (95% CIs) from Cox proportional hazards regressions in the total population (*n* = 340,152) and in never smokers (*n* = 160,111); *n* number of cancer incidence cases, CNS central nervous system, SCC squamous cell carcinomas.

The HR for overall cancer risk per 1 SD increment in PC1 was 1.07 (95% CI = 1.05–1.08) (Fig. 2). In cancer type-specific analyses, a 1 SD increment in PC1 was associated with increased risks for malignant tumours of the corpus uteri, oesophagus (adeno), liver, kidney, gallbladder, colon, pancreas, myeloma, breast (postmenopausal), and rectum. An inverse relationship was observed between PC1 and cancers of the prostate and oesophagus (SCC). All these associations passed the Bonferroni-corrected *P* ≤ 0.001. Among never smokers, these estimates remained largely unchanged, except for smoking-related cancers, where the point estimate increased (lips, oral cavity, pharynx: 1.13; 0.98–1.30) or showed a tendency towards the null (lung, larynx, oesophagus (SCC)).

### Results for PC2 (tall stature; low WHR)

**Fig. 3:**
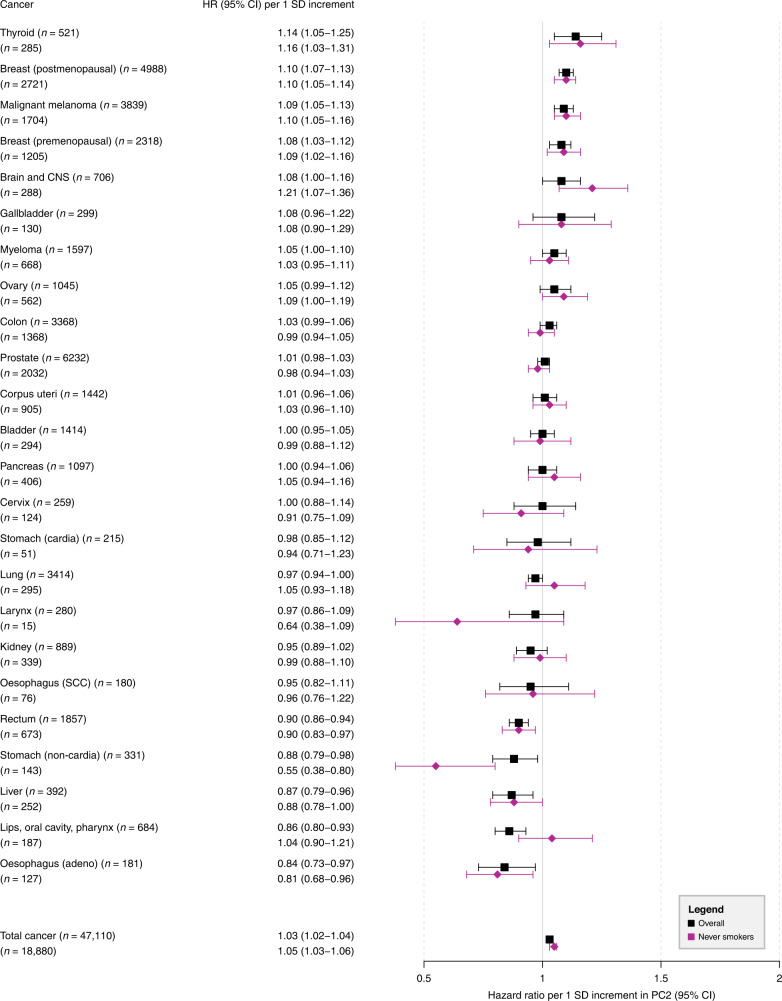
Hazard ratios (HRs) for total cancer and 24 cancer subtypes per 1 SD increment in the second principal component (PC2; tall stature, low waist-to-hip ratio). HRs with corresponding 95% confidence intervals (95% CIs) from Cox proportional hazards regressions in the total population (*n* = 340,152) and in never smokers (*n* = 160,111); *n* number of cancer incidence cases, CNS central nervous system, SCC squamous cell carcinomas.

The association between PC2 and overall cancer showed a slightly increased risk per 1 SD increment (HR = 1.03; 95% CI = 1.02–1.04) (Fig. 3). Positive associations were observed for cancers of the thyroid, breast (post- and premenopausal) and malignant melanoma. All these associations passed the Bonferroni-corrected *P* ≤ 0.001, except thyroid cancer (*P* = 0.003). An inverse relationship was observed for tumours of the rectum and lips, oral cavity, pharynx. Inverse associations were also observed for cancers of the stomach (non-cardia), liver, and oesophagus (adeno), but these associations did not pass the Bonferroni-corrected *P* ≤ 0.001. When these analyses were repeated among never smokers, the point estimates remained largely unchanged, except for a stronger positive association for cancers of the brain and CNS, no association for cancers of the lips, oral cavity, pharynx, and a stronger inverse association for non-cardia stomach cancer.

### Results for PC3 (tall stature; high WHR)

**Fig. 4:**
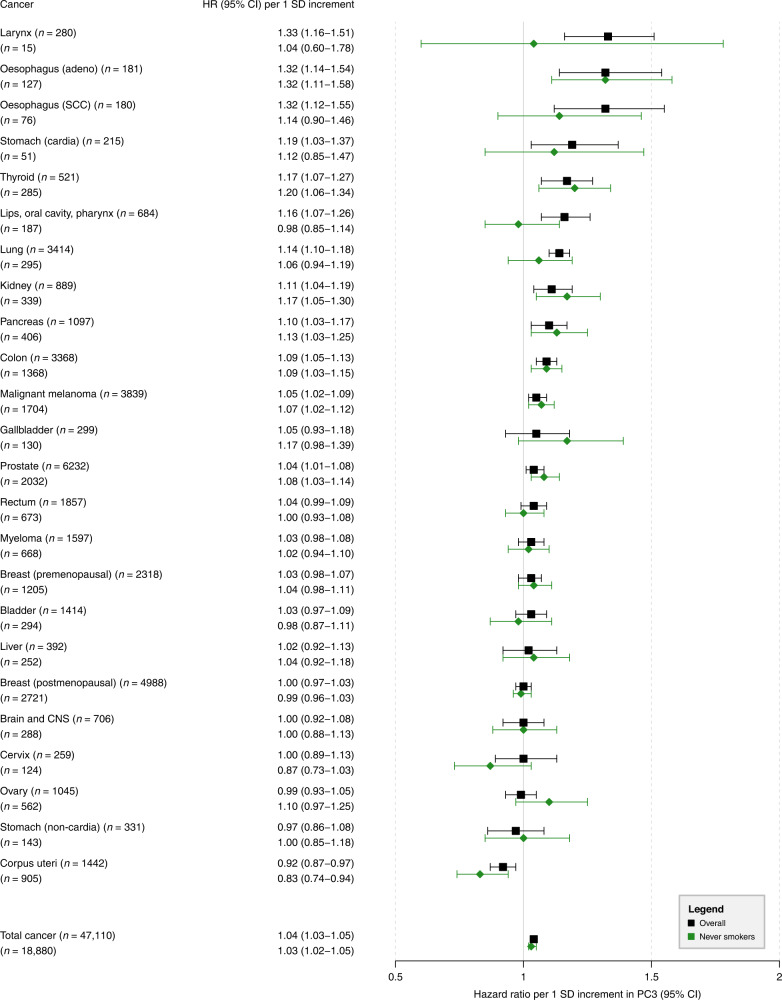
Hazard ratios (HRs) for total cancer and 24 cancer subtypes per 1 SD increment in the third principal component (PC3; tall stature, high waist-to-hip ratio). HRs with corresponding 95% confidence intervals (95% CIs) from Cox proportional hazards regressions in the total population (*n* = 340,152) and in never smokers (*n* = 160,111); *n* number of cancer incidence cases, CNS central nervous system, SCC squamous cell carcinomas.

PC3 was positively associated with overall cancer risk, with an HR of 1.04 (95% CI = 1.03–1.05) per 1 SD increment (Fig. 4). Positive associations were observed for 12 of 24 different cancers, of which 8 also passed the Bonferroni-corrected *P* ≤ 0.001 (Supplementary Table 7). However, among never smokers, associations with five of these cancer types were substantially attenuated (larynx, oesophageal SCC, stomach cardia, lips, oral cavity, pharynx, and lung). An inverse association was found for cancer of the corpus uteri (*P* < 0.001), with a more pronounced inverse association among never smokers.

### Results for PC4 (high BMI and weight; low WC and HC)

There was no association between PC4 and overall cancer risk (HR = 1.00; 95% CI = 0.99–1.01) (Supplementary Fig. 8). A relatively robust positive association was observed with thyroid cancer risk (HR = 1.10, 95% CI = 1.00–1.21), which however did not pass the Bonferroni-corrected *P* ≤ 0.001.

### Sensitivity analyses

After excluding the first 2 years of follow-up, the point estimates for PC1 remained largely unchanged, except for cervical cancer, for which the HR decreased (Supplementary Table 5). There was also little change for PC2, except for an even lower risk for laryngeal cancer (Supplementary Table 6). For PC3 and PC4, no sizeable changes in the associations were observed (Supplementary Tables 7 and 8).

In analyses stratified by age, most HRs were largely consistent across the two age groups (<52.3 vs. ≥52.3 years). Exceptions were as follows. For PC1, HRs for cancers of the pancreas and thyroid were stronger in the younger as compared to the older age group (Supplementary Table 5). For PC2, the HR for gallbladder cancer was stronger in the younger as compared to older age group, whereas HRs were less strong for cancers of the brain and CNS, breast (premenopausal), and thyroid (Supplementary Table 6). For PC3, HRs for oesophageal (adeno and SCC), laryngeal, stomach (cardia), and thyroid cancers were stronger in the younger as compared to the older age group (Supplementary Table 7). For PC4, a positive association was observed with liver cancer in the younger age group, while this association was inverse in the older age group (Supplementary Table 8).

In a further analysis, BMI (per 5 kg/m^2^ increment = 1 SD) was positively associated with risk of cancers of the corpus uteri, oesophagus (adeno), kidney, thyroid, gallbladder, breast (postmenopausal), colon, pancreas, rectum, and multiple myeloma (Supplementary Fig. 9). Positive associations were also seen for tumours of the stomach (cardia) and ovary, but confidence intervals included the null. An inverse relationship was found for seven cancer types, including cancers of the oesophagus (SCC), cervix, lung, lips, oral cavity, pharynx, stomach (non-cardia), larynx, and malignant melanoma. After restricting the analyses to never smokers, BMI remained inversely associated only with malignant melanoma.

## Discussion

PCs of six anthropometric traits that capture four distinct body shape phenotypes were differentially associated with the risk of overall cancer and 17 site-specific cancers. Some novel associations were identified, including a positive relation of PC3 with lung cancer, oesophageal SCC, and malignant melanoma. Furthermore, PC1 and/or PC3 were positively associated with hepatobiliary cancers, malignant melanoma, and total prostate cancer, while BMI was unrelated to those cancers (Supplementary Fig. 9). These findings suggest that the current cancer burden associated with adiposity and body size based on classical anthropometric traits is likely underestimated. Leveraging information from multiple anthropometric traits may better capture the heterogeneous expression of adiposity and its health consequences than BMI.

We showed that these body shape phenotypes are congruent with Ried et al. [16] and thus stable across study populations (Fig. 1) and between men and women (Supplementary Tables 2 and 3). The PCs may represent body shape phenotypes more holistically as compared to single anthropometric traits due to the way they combine. However, their interpretation is less straightforward. To circumvent this difficulty, we provided the arithmetic means of each anthropometric trait among participants in the top and bottom 5% percentile across the four body shape phenotypes together with the population variation (1 SD) of these traits and integrated this information in Supplementary Figs. 3–6 (example in men). For PC1, the difference in height between the top and bottom 5% percentile was 7 cm, which corresponded to 1 SD for height in the study population. In contrast, the difference in BMI was 13.1 kg/m^2^, which was equal to 3.6 SD (13.1 kg/m^2^ over population SD for BMI of 3.6 kg/m^2^); similarly pronounced differences were observed for weight, WC, and HC, but not WHR. This means that with increments in loadings of PC1, BMI, weight, WC, and HC increased by much more than height and WHR. With increments in PC2, we observed a 3.3 SD increment in height and a ~1 SD increment in weight and HC, while WHR, WC, and BMI decreased by ≤1 SD. With increments in PC3, we observed a 2 SD increment in height and ~1 SD increments in WC, WHR, and weight, while BMI and HC remained similar. PC3 could thus indicate a tall and centrally obese phenotype. With increments in PC4, BMI, weight, and WHR increased by ≥1 SD while height, WC, and HC remained similar.

In a post hoc analysis, we calculated median loadings for the body shape phenotypes by smoking status. A pronounced difference was observed for PC3, where current smokers, as compared to never smokers, had higher loadings on PC3 indicating a propensity towards central adiposity for the same level of BMI (Supplementary Table 9). Tobacco smoking, and lifestyle behaviours in general, may play an important role in shaping these phenotypes. Differences in body composition, especially different proportions of muscle mass and visceral adipose tissue across body shape phenotypes, and how these are influenced by lifestyle factors should be investigated in future studies.

### PC1 and cancer

The results of PC1, a body shape characterised by general obesity, confirm previous findings on the association between excess body fat and cancer risk and are also in line with previous studies that have considered BMI as a risk factor [2, 29]. We observed positive associations for all established obesity-related cancers. Inverse relationships for cancers of the prostate, larynx, and oesophagus (SCC) are also consistent with findings from a large Spanish cohort [29]. In contrast to previous studies, we found a strong positive association with liver cancer but no association with BMI. The lack of association with BMI suggests that PC1 captures phenotype information beyond that provided by BMI.

### PC2 and cancer

Our results for PC2 (tall with low WHR vs. short with high WHR) are not directly comparable with previous studies given the specificity of this body shape. There is nevertheless some overlap between our findings and the literature. There is strong evidence that adult attained height increases the risk of cancers of the premenopausal and postmenopausal breast, skin, colorectum, endometrium, prostate, ovaries, pancreas, kidney, and possibly liver [7–12]. This is congruent with our findings except for liver cancer, where we found a relatively robust inverse association, although this association (*P* = 0.007) did not pass the more stringent multiple-testing corrected *P*-value of 0.001. Height may represent a surrogate measure for cancer risk factors early in life [30]. Potential aetiologic mechanisms linking taller height to an increased cancer risk include more stem cells with an increased number of mutations during cell division, and insulin-like growth factor 1, which is a major determinant of height and organ size and of cancer risk [13, 14].

In addition to liver cancer, we also found robust inverse associations between PC2 and cancers of the rectum, stomach (non-cardia), and oesophagus (adeno). Non-cardia gastric cancer is caused mainly by *Helicobacter pylori* infections, which are associated with growth retardation in children [31]. Our finding of an inverse relation with non-cardia gastric cancer is supported by the observation of that cancer being likely to occur in individuals of short stature. A Mendelian randomisation analysis of adult height found an inverse association with oesophageal cancer but a weak positive association with liver cancer [32].

### PC3 and cancer

PC3 (tall height; high WHR) represents abdominal obesity in combination with height. Abdominal obesity poses risk for oesophageal adenocarcinoma [33], consistent with our findings. Mechanistically, this could be explained by gastroesophageal reflux disease (GERD) predisposing to Barrett’s oesophagus [34]. Robust positive associations were also observed for cancers of the thyroid, kidney, pancreas, colon, and prostate cancers for which abdominal adiposity and attained height have been implicated as risk factors [7]. A positive association was observed between PC3 and malignant melanoma, indicating attained height as a key anthropometric risk factor. This is supported by a similarly strong positive association between PC2 (tall height, low WHR) and malignant melanoma.

PC3 also showed strong positive associations with smoking-related cancers including larynx, oesophageal SCC, oral cancers and pharynx, and lung. Notably, among never smokers these associations were completely attenuated for cancers of the larynx, oral cancers and pharynx suggesting residual confounding by smoking. Associations with the risk of oesophageal SCC and lung cancer were also attenuated among never smokers but remained imprecisely positively associated. There is some evidence that height is positively associated with the risk of oesophageal SCC and lung cancer [9]. However, further studies are required to corroborate these findings. The observed inverse association between PC3 and corpus uteri cancer is striking. One hypothesis is that HC tracks with gluteofemoral fat accumulation, which is associated with a more favourable adipokine profile and increased lipoprotein lipase activity [35], profiles that have been linked to lower endometrial cancer risk [36].

### PC4 and cancer

PC4 only explained a small proportion (3%) of the overall variation in anthropometric traits. Nevertheless, it may represent a rare phenotype of potential relevance in the aetiology of certain cancers. Ried et al. identified two genetic loci for PC4 that have not previously been captured by single-trait anthropometric GWAS [16]. One of these two single-nucleotide polymorphisms has previously been associated with increased levels of circulating adiponectin [16], which has been implicated in cancer development [37]. In our analysis, we found a relatively robust positive association with thyroid cancer (Supplementary Fig. 8 and Supplementary Table 8). Whether altered levels of adiponectin plays a functional role in thyroid cancer in addition to or independently of excess weight is currently unclear [38].

### Strengths and limitations

The primary strength of our study is the novel results on body shape phenotypes in relation to cancer incidence. Further assets are the large number of cases, extensive follow-up time, and inclusion of participants from different European countries. In addition, we conducted various informative sub-analyses to rule out the influence of residual confounding and reverse causality. Limitations are potential selection bias with health-conscious individuals being over-represented, and the study being restricted to Caucasian ethnicities. Furthermore, a one-time measure of body shape assumes that participants do not change their exposure profile during follow-up, which is a strong assumption. However, at least for BMI, we show in yet unpublished work that baseline BMI compared to cumulative BMI yielded comparable risk estimates across 26 cancer types (Recalde et al., Nat Commun, provisionally accepted). We also note that there was no interaction between the body shape phenotypes and the time scale associated with cancer risk in our analysis. Taken together, baseline body shape phenotypes very likely provide a good approximation of long-term exposure.

## Conclusion

In this multi-national study, distinct body shape phenotypes were positively associated with risks of 17 different cancers. Several entirely novel relationships were identified that have thus far remained undetected in previous studies using classical anthropometric traits. Derived body shapes may reveal underlying biological pathways, thereby providing new insights into cancer development. Such knowledge could help inform cancer prevention strategies.

## Disclaimer

Where authors are identified as personnel of the International Agency for Research on Cancer/World Health Organisation, the authors alone are responsible for the views expressed in this article and they do not necessarily represent the decisions, policy or views of the International Agency for Research on Cancer/World Health Organisation.

## Supplementary information

STROBE checklist

Supplementary materials

## Data Availability

EPIC data are available for investigators who seek to answer important questions on health and disease in the context of research projects that are consistent with the legal and ethical standard practices of IARC/WHO and the EPIC Centres. For information on how to submit an application for gaining access to EPIC data and/or biospecimens, please follow the instructions http://epic.iarc.fr/access/index.php.
