# Supplementary material for: Body shape phenotypes of multiple anthropometric traits and cancer risk: a multi-national cohort study
Source: Br J Cancer. 2022 Dec 2;128(4):594–605. doi: 10.1038/s41416-022-02071-3 (PMC9938222; doi:10.1038/s41416-022-02071-3)
Supplement: Supplementary file 2 — Supplementary materials [file 41416_2022_2071_MOESM2_ESM.docx]

## Supplementary materials

Supplementary Figure 1: Flow chart of the EPIC study population.

Supplementary Table 1: List of codes used to define cancer cases according to the International Classification of Diseases for Oncology, 3rd edition.

| **Cancer type** | **Topography according to ICD-O-3** |
| --- | --- |
| Bladder | C65-C68 |
| Brain and CNS | C70-C72 |
| Breast | C50 |
| Cervix | C53 |
| Colon | C18 |
| Corpus uteri | C54-C55 |
| Esophagus (adeno) | C15 |
| Esophagus (SCC) | C15 |
| Gallbladder | C23-C24 |
| Kidney | C64 |
| Larynx | C32 |
| Lip, Oral cavity, Pharynx | C00-C14 |
| Liver | C22 |
| Lung | C33-C34 |
| Melanoma | C44 |
| Myeloma | C42 |
| Ovary | C56 |
| Pancreas | C25 |
| Prostate | C61 |
| Rectum | C19-C20 |
| Stomach (cardia) | C16.0 |
| Stomach (non-cardia) | C16.1-C16.6 |
| Thyroid | C73 |

ICD-O-3: International Classification of Diseases for Oncology, 3rd edition; CNS: central nervous system; CNS: central nervous system; SCC: squamous cell carcinomas.

### Assessment of covariates

At baseline, information on socioeconomic and lifestyle factors, dietary intake, and medical information was collected using validated questionnaires (1). Information on education, smoking status and intensity, alcohol consumption, and menopausal status was also obtained. Participants' physical activity was estimated using questions related to physical activity in the past year in the domains of work, leisure, and home. This information was used to derive the previously validated Cambridge Physical Activity Index (2). To obtain information on eating habits of the participants, validated country- or center-specific dietary questionnaires were used. From these, the Mediterranean Diet Score, an indicator of diet quality, was derived (3). Potential confounding variables were identified a priori using Directed Acyclic Graphs (DAGs) (4, 5). For the selection of confounding variables, the DAG was drawn for the present analyses using the DAGitty web application (6) (eFigure 2). The multivariable models included education (none, primary school completed, technical/professional school, secondary school, longer education (including university degree)), smoking status/intensity (never, quit ≤10 years ago, quit 11-20 years ago, quit >20 years ago, currently 1-15 cigarettes/day, currently 16-25 cigarettes/day, currently ≥26 cigarettes/day, currently pipe/cigar, occasional), physical activity (inactive, moderately inactive, moderately active, active), alcohol consumption (g/day), and healthy diet represented by the Mediterranean Diet Score (low, medium, high).


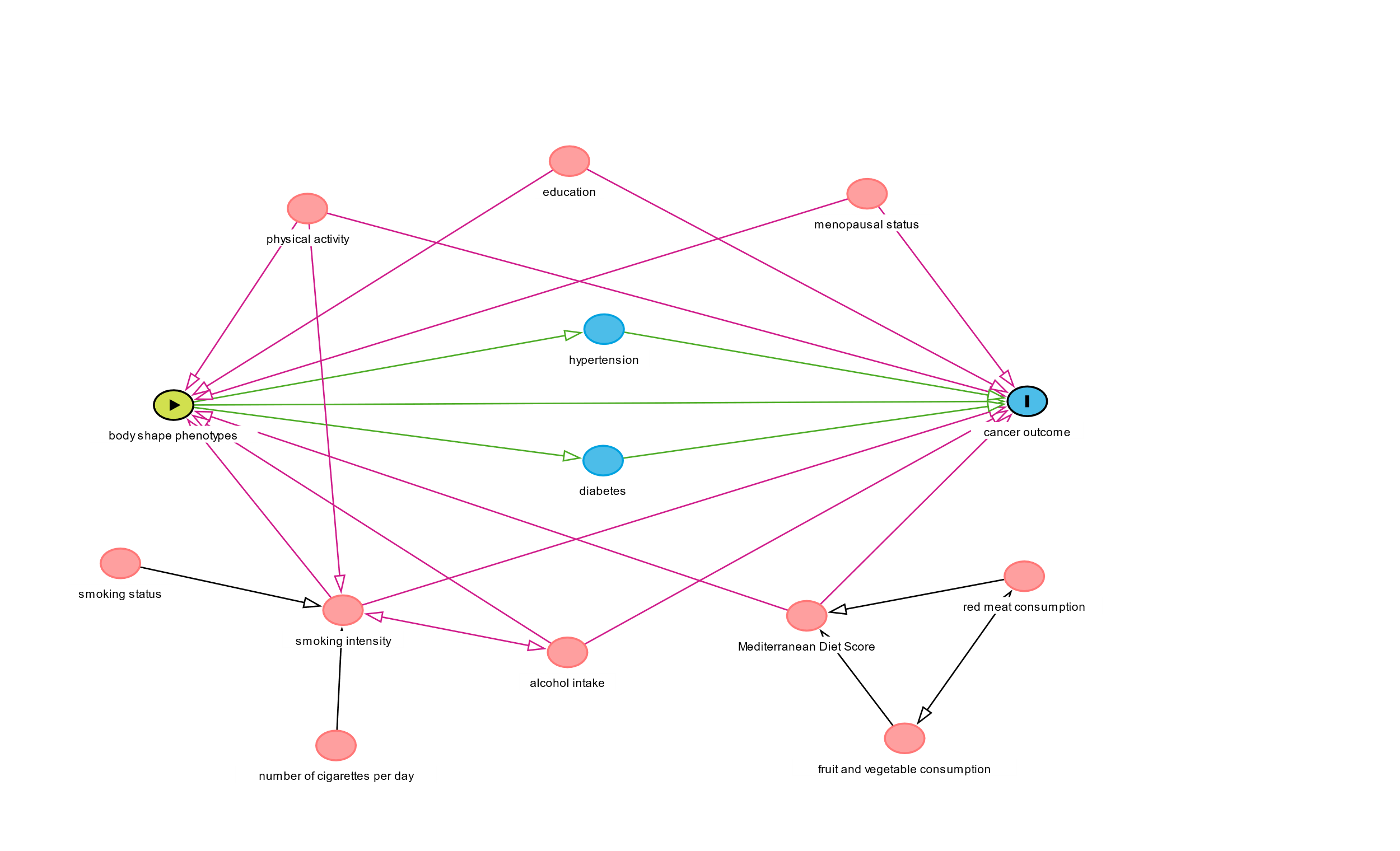


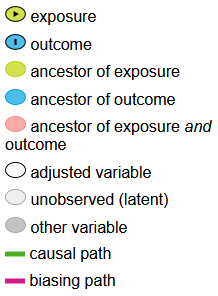

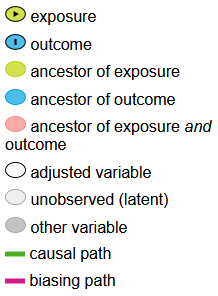


Supplementary Figure 2: Directed Acyclic Graph showing the relationship between potential confounders in the association between body shape phenotypes and cancer risk.

Supplementary Table 2: Loadings and explained variance of the principal components for men (n=118,218).

|  | **PC1** | **PC2** | **PC3** | **PC4** | **PC5** | **PC6** |
| --- | --- | --- | --- | --- | --- | --- |
| Height | 0.131 | 0.798 | 0.466 | -0.058 | 0.306 | -0.179 |
| Weight | 0.482 | 0.211 | -0.031 | -0.493 | -0.597 | 0.351 |
| BMI | 0.463 | -0.216 | -0.302 | -0.503 | 0.547 | -0.31 |
| Waist circumference | 0.492 | -0.128 | 0.131 | 0.415 | -0.375 | -0.642 |
| Hip circumference | 0.431 | 0.240 | -0.455 | 0.565 | 0.235 | 0.418 |
| WHR | 0.330 | -0.446 | 0.682 | 0.092 | 0.235 | 0.403 |
| Explained variance [%] | 64.21 | 20.33 | 12.02 | 3.35 | 0.06 | 0.04 |

BMI: body mass index; PC: principal component; WHR: waist-to-hip ratio.

Supplementary Table 3: Loadings and explained variance of the principal components for women (n=221,934).

|  | **PC1** | **PC2** | **PC3** | **PC4** | **PC5** | **PC6** |
| --- | --- | --- | --- | --- | --- | --- |
| Height | 0.069 | 0.787 | 0.537 | 0.029 | -0.052 | 0.291 |
| Weight | 0.489 | 0.228 | -0.046 | 0.486 | 0.109 | -0.677 |
| BMI | 0.481 | -0.112 | -0.290 | 0.479 | -0.109 | 0.656 |
| Waist circumference | 0.492 | -0.158 | 0.188 | -0.413 | -0.716 | -0.122 |
| Hip circumference | 0.454 | 0.224 | -0.347 | -0.602 | 0.505 | 0.076 |
| WHR | 0.277 | -0.491 | 0.685 | 0.021 | 0.454 | 0.076 |
| Explained variance [%] | 62.75 | 19.33 | 15.25 | 2.55 | 0.06 | 0.06 |

BMI: body mass index; PC: principal component; WHR: waist-to-hip ratio.


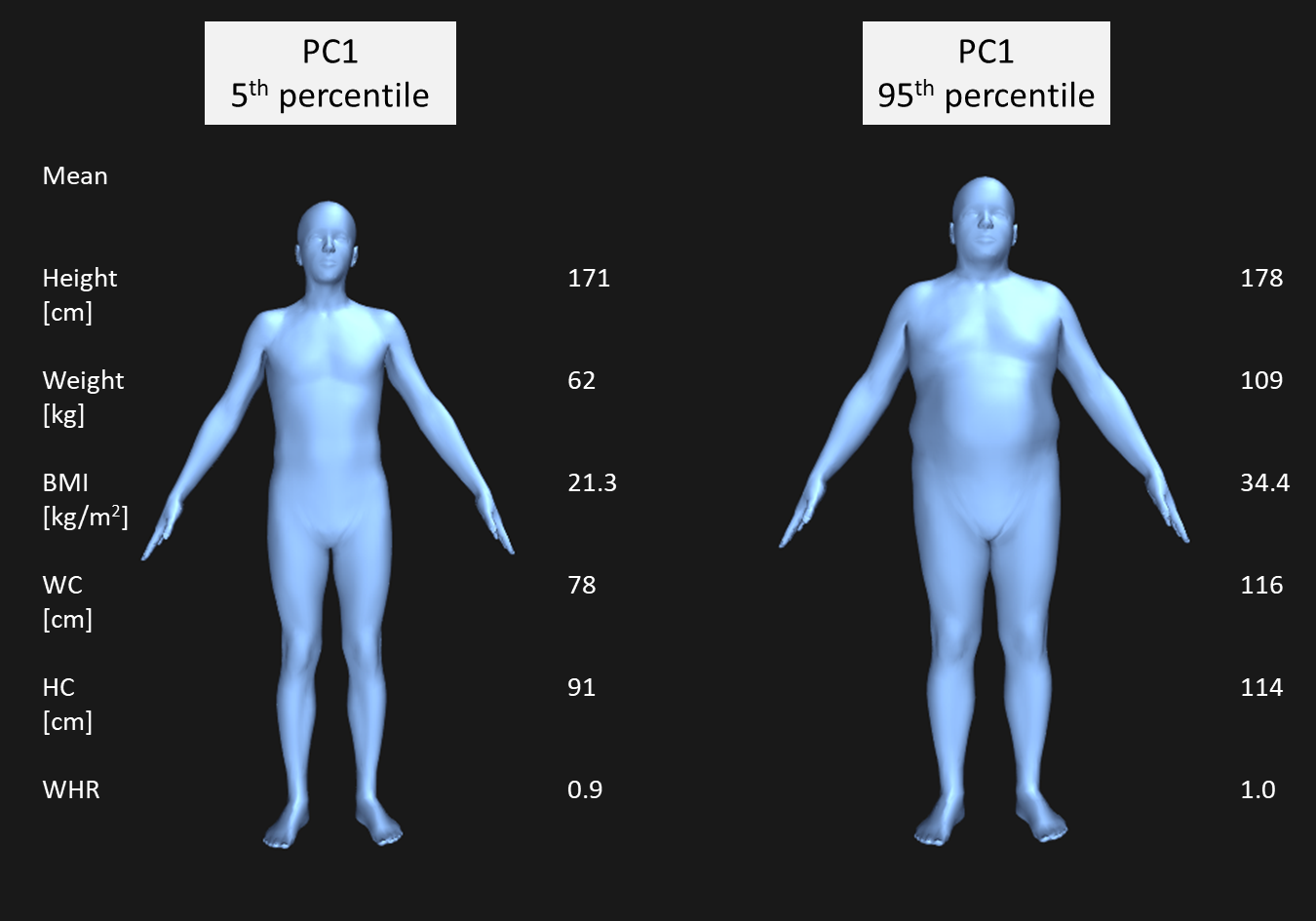


Supplementary Figure 3: Visualization of body shapes for the first principal component (PC1).

For the plot, mean values of anthropometric data of the top and bottom 5% of the EPIC study population were calculated for PC1 (example: men); <https://bodyvisualizer.com/>.

For better interpretation, we provide the population standard deviation of each of the six anthropometric measures in men: height [cm] 7.2; weight [kg] 12.0; BMI [kg/m^2^] 3.6; WC [cm] 10.1; HC [cm] 6.8; WHR 0.1.


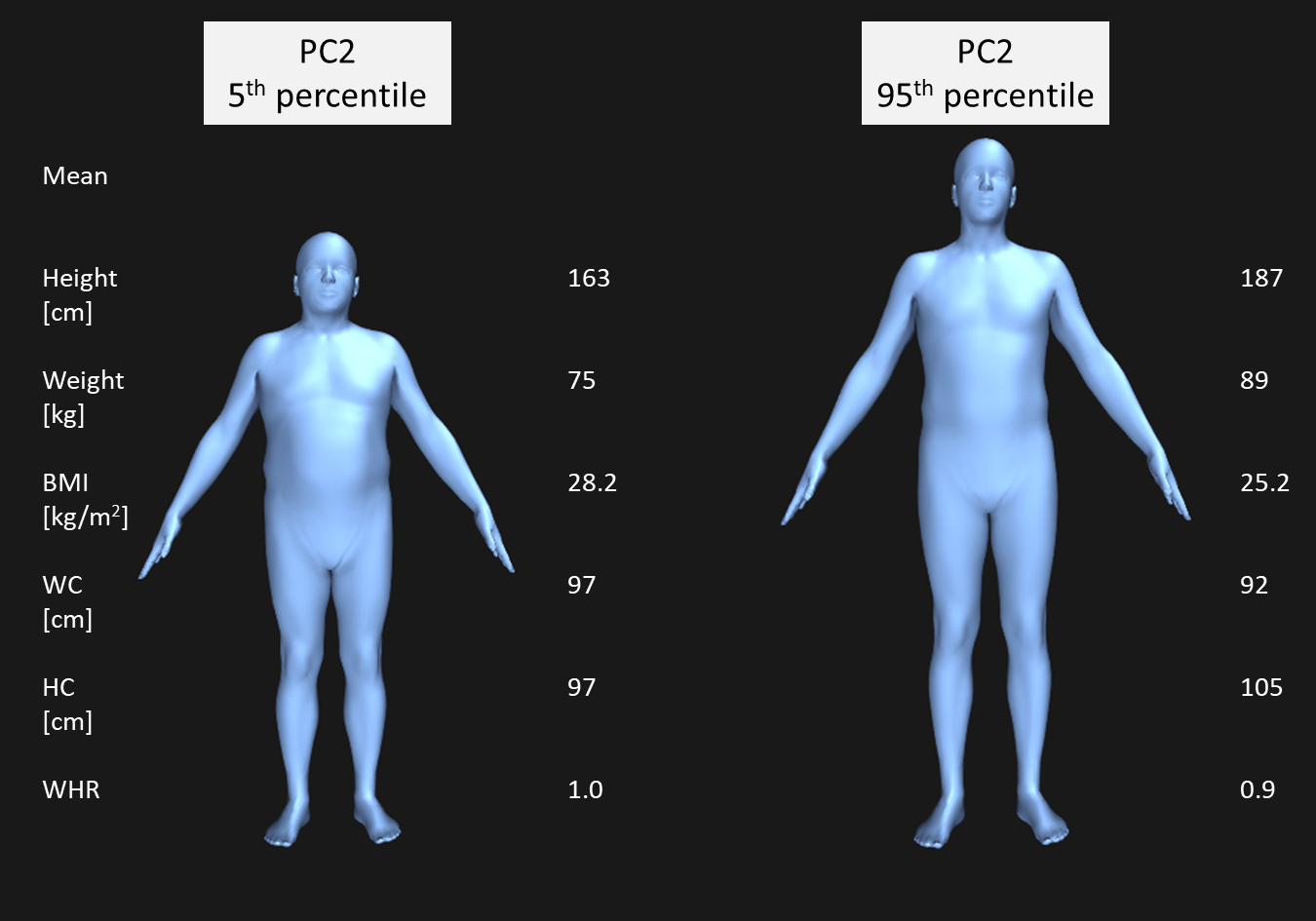


Supplementary Figure 4: Visualization of body shapes for the second principal component (PC2).

For the plot, mean values of anthropometric data of the top and bottom 5% of the EPIC study population were calculated for PC2 (example: men); <https://bodyvisualizer.com/>.

For better interpretation, we provide the population standard deviation of each of the six anthropometric measures in men: height [cm] 7.2; weight [kg] 12.0; BMI [kg/m^2^] 3.6; WC [cm] 10.1; HC [cm] 6.8; WHR 0.1.


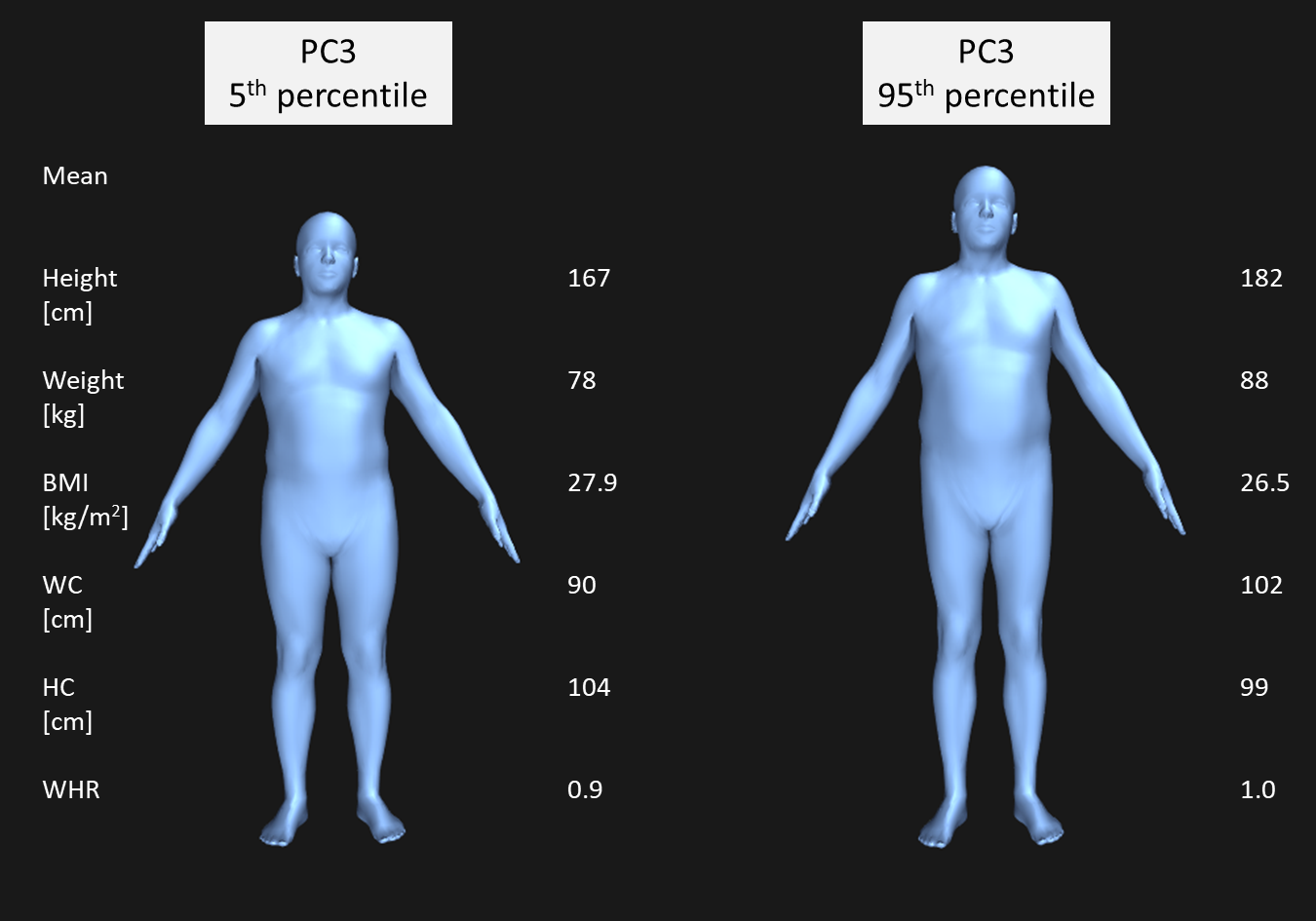


Supplementary Figure 5: Visualization of body shapes for the third principal component (PC3).

For the plot, mean values of anthropometric data of the top and bottom 5% of the EPIC study population were calculated for PC3 (example: men); https://bodyvisualizer.com/.

For better interpretation, we provide the population standard deviation of each of the six anthropometric measures in men: height [cm] 7.2; weight [kg] 12.0; BMI [kg/m^2^] 3.6; WC [cm] 10.1; HC [cm] 6.8; WHR 0.1.


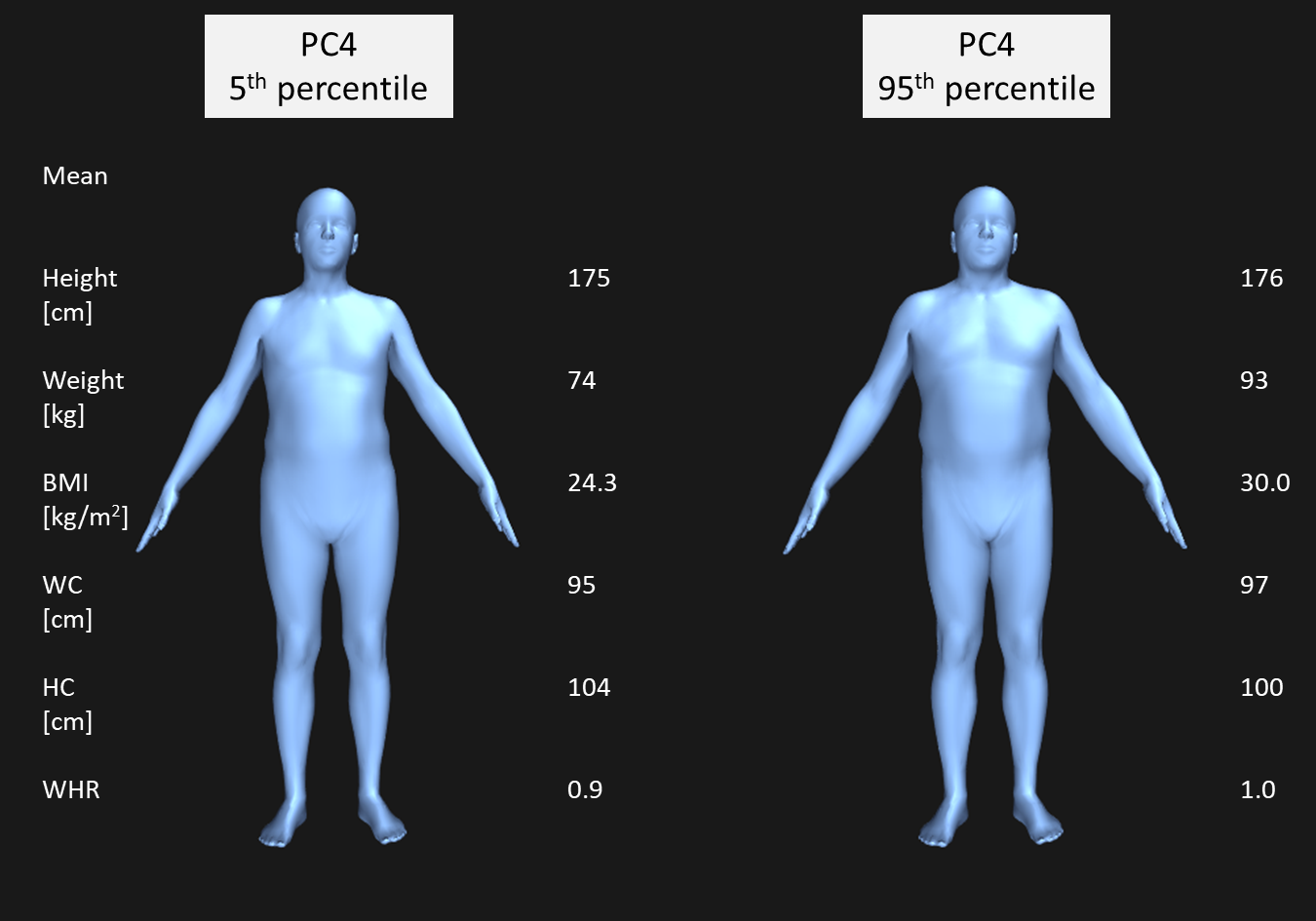


Supplementary Figure 6: Visualization of body shapes for the fourth principal component (PC4).

For the plot, mean values of anthropometric data of the top and bottom 5% of the EPIC study population were calculated for PC4 (example: men); https://bodyvisualizer.com/.

For better interpretation, we provide the population standard deviation of each of the six anthropometric measures in men: height [cm] 7.2; weight [kg] 12.0; BMI [kg/m^2^] 3.6; WC [cm] 10.1; HC [cm] 6.8; WHR 0.1.

Supplementary Figure 7: Correlation matrix showing Pearson’s correlation coefficients for the four principal components (PCs) and the six anthropometric measures body mass index (BMI), weight, height, waist (WC) and hip circumferences (HC), and waist-to-hip ratio (WHR).


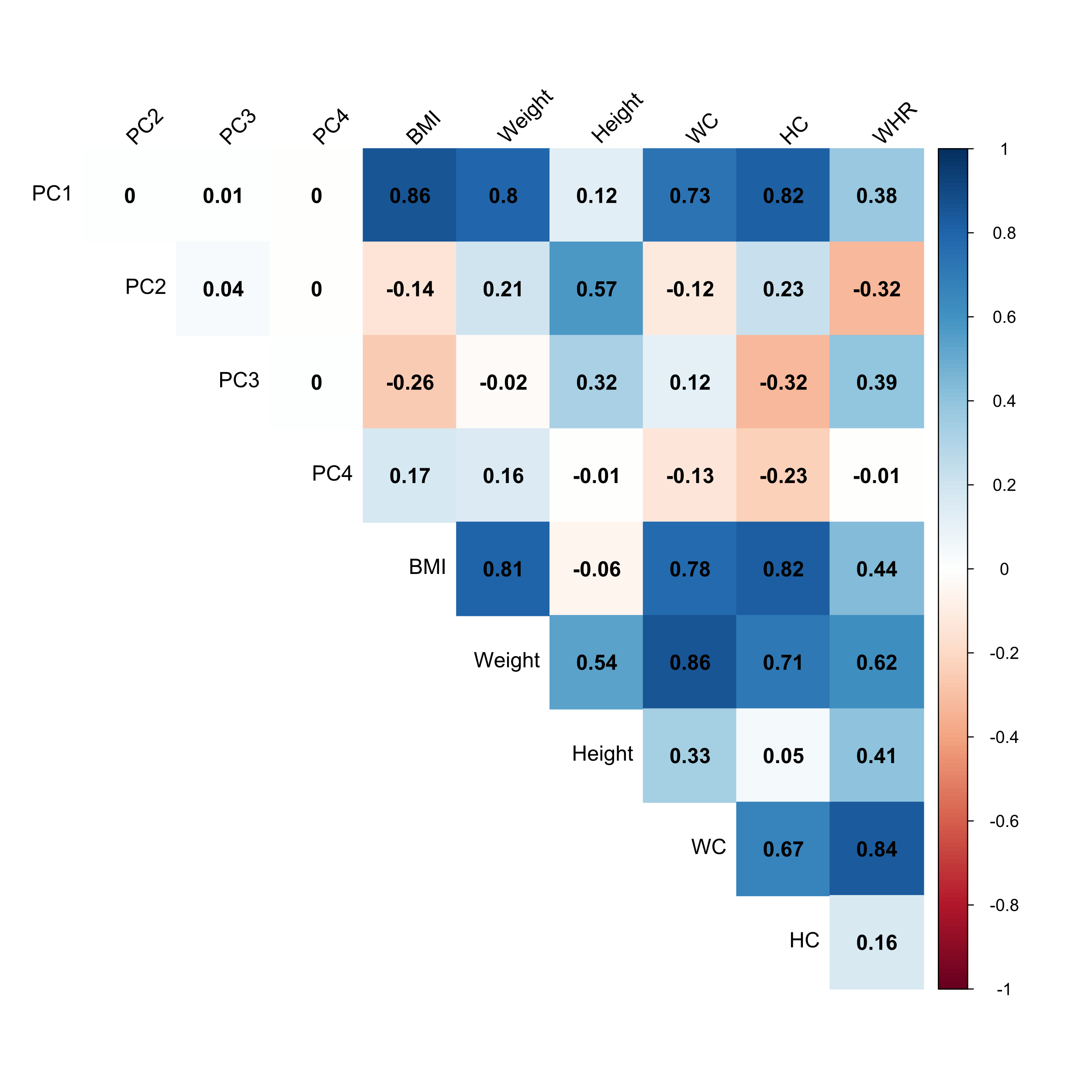


Supplementary Table 4: Sex- and country-specific characteristics of study participants.

| **Country** | **Number (n)** | **Total cancer cases (n)** | **Age (years)** | **Median follow-up time (years)** | **Weight (kg)** | **Height (cm)** | **BMI (kg/m^2)^** | **Waist circumference (cm)** | **Hip circumference (cm)** | **Waist-to-hip-ratio** |  |
| --- | --- | --- | --- | --- | --- | --- | --- | --- | --- | --- | --- |
| **Men (mean and SD)** | | | | | | | | | | | |
| Italy | 14,010 | 1706 | 50.2 (7.5) | 14.7 | 77.7 (11.0) | 171.7 (6.7) | 26.4 (3.3) | 92.5 (9.0) | 99.3 (6.4) | 0.93 (0.06) |  |
| Spain | 15,059 | 2313 | 50.7 (7.2) | 16.8 | 81.2 (10.8) | 169.0 (6.3) | 28.4 (3.4) | 99.4 (9.0) | 105.0 (6.6) | 0.95 (0.06) |  |
| United Kingdom | 21,895 | 3383 | 52.7 (13.7) | 15.8 | 78.1 (11.6) | 175.5 (6.8) | 25.4 (3.4) | 90.9 (9.2) | 99.5 (5.8) | 0.91 (0.06) |  |
| The Netherlands | 9592 | 853 | 43.2 (11.0) | 15.2 | 81.1 (12.1) | 178.5 (7.3) | 25.5 (3.5) | 90.5 (10.8) | 99.4 (6.6) | 0.91 (0.07) |  |
| Germany | 21,168 | 2420 | 52.4 (7.6) | 11.6 | 83.0 (12.1) | 175.4 (6.7) | 27.0 (3.6) | 95.5 (10.2) | 101.1 (6.8) | 0.94 (0.06) |  |
| Sweden | 10,241 | 3072 | 59.0 (7.0) | 18.4 | 80.8 (12.1) | 176.4 (6.6) | 25.9 (3.4) | 93.7 (10.0) | 99.2 (7.0) | 0.94 (0.06) |  |
| Denmark | 26,253 | 5844 | 56.6 (4.4) | 16.2 | 83.0 (12.3) | 176.7 (6.5) | 26.6 (3.6) | 96.0 (9.9) | 100.4 (6.7) | 0.96 (0.06) |  |
| **Total** | 118,218 | 19,591 | 52.7 (9.6) | 15.4 | 80.9 (12.0) | 174.8 (7.2) | 26.5 (3.6) | 94.3 (10.1) | 100.6 (6.8) | 0.94 (0.06) |  |
| **Women (mean and SD)** | | | | | | | | | | | |
| France | 19,029 | 2179 | 52.8 (6.5) | 14.8 | 60.4 (10.1) | 160.8 (5.8) | 23.4 (3.7) | 76.6 (9.5) | 98.3 (8.2) | 0.78 (0.07) |  |
| Italy | 30,466 | 3323 | 50.6 (8.1) | 15.1 | 64.4 (11.0) | 158.5 (6.1) | 25.7 (4.3) | 80.0 (10.6) | 100.4 (8.7) | 0.80 (0.07) |  |
| Spain | 24,657 | 2130 | 48.3 (8.4) | 16.7 | 69.0 (11.3) | 156.8 (5.8) | 28.1 (4.7) | 87.3 (11.2) | 105.8 (9.5) | 0.82 (0.06) |  |
| United Kingdom | 50,916 | 5867 | 47.4 (14.3) | 16.1 | 64.4 (11.3) | 163.3 (6.2) | 24.2 (4.2) | 74.7 (9.4) | 97.9 (8.0) | 0.76 (0.06) |  |
| The Netherlands | 26,753 | 3407 | 51.0 (11.6) | 15.0 | 68.3 (11.5) | 164.9 (6.4) | 25.1 (4.1) | 80.7 (10.6) | 102.2 (8.5) | 0.79 (0.07) |  |
| Germany | 27,365 | 2180 | 49.1 (9.0) | 11.5 | 68.2 (12.5) | 163.3 (6.2) | 25.6 (4.6) | 80.8 (11.6) | 101.4 (9.3) | 0.80 (0.07) |  |
| Sweden | 14,070 | 2970 | 57.3 (7.9) | 18.3 | 67.0 (11.5) | 163.6 (6.1) | 25.0 (4.2) | 77.8 (10.4) | 97.9 (9.5) | 0.79 (0.05) |  |
| Denmark | 28,678 | 5463 | 56.7 (4.4) | 16.4 | 68.8 (12.1) | 164.2 (6.0) | 25.6 (4.4) | 82.0 (11.2) | 101.7 (8.8) | 0.81 (0.07) |  |
| **Total** | 221,934 | 27,519 | 50.9 (10.5) | 15.3 | 66.2 (11.7) | 162.0 (6.7) | 25.3 (4.5) | 79.6 (11.2) | 100.6 (9.1) | 0.79 (0.07) |  |

BMI: body mass index; n: number; SD: standard deviation.


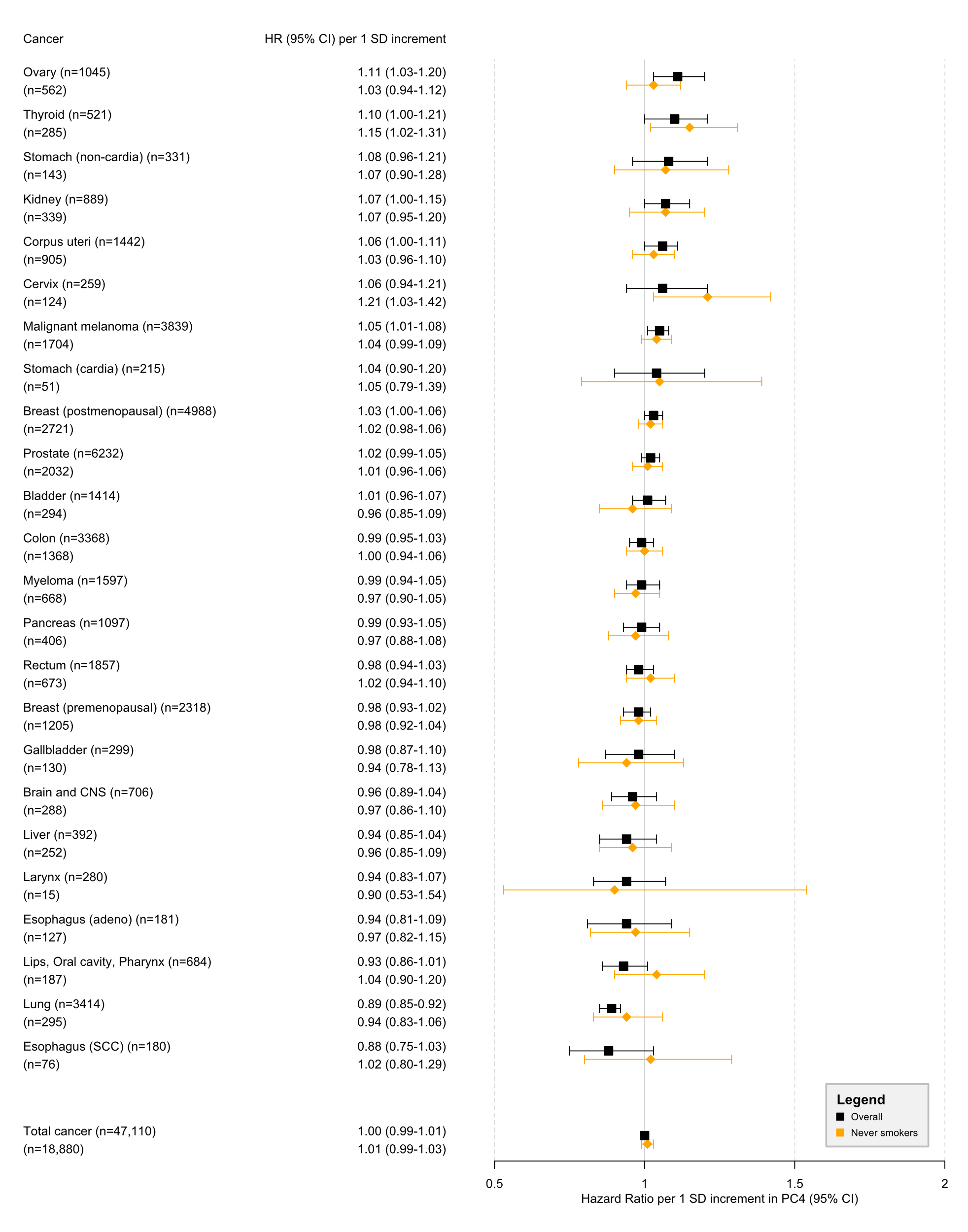


Supplementary Figure 8: Hazard ratios (HRs) for total cancer and 24 cancer subtypes per 1 SD increment in the fourth principal component (PC4; high body mass index and weight; low waist and hip circumference). HRs with corresponding 95% confidence intervals (95% CIs) from Cox proportional hazards regressions in the total population (n=340,152) and in never smokers (n=160,111); n: number of cancer incidence cases; CNS: central nervous system; SCC: squamous cell carcinomas.

Supplementary Table 5: Relations of principal component 1 (overall adiposity) per 1 SD increment to cancer incidence in the complete case dataset (n=340,152), after further excluding the initial 2 years of follow-up (n=334,605), in current smokers (n=79,819), fitted as a crude model (n=340,152)^1^, in participants younger than 52.3 years (n=169,473), and older than 52.3 years (n=170,679).

|  | **Cancer incidence** | | | **Cancer incidence** | | **Cancer incidence** | | **Cancer incidence** | | **Cancer incidence** | | **Cancer incidence** | |
| --- | --- | --- | --- | --- | --- | --- | --- | --- | --- | --- | --- | --- | --- |
|  | Complete case dataset | | | FU>2 years | | Current smokers | | Crude model^2^ | | Younger <52.3 years | | Older ≥52.3 years | |
| **Principal component 1** | HR (95% CI) | n | *P* | HR (95% CI) | n | HR (95% CI) | n | HR (95% CI) | n | HR (95% CI) | n | HR (95% CI) | n |
| Bladder | 1.03  (0.98-1.09) | 1414 | 0.287 | 1.03  (0.97-1.09) | 1327 | 0.98  (0.90-1.07) | 608 | 1.03  (0.97-1.09) | 1414 | 1.09  (0.96-1.23) | 279 | 1.01  (0.95-1.08) | 1135 |
| Brain and CNS | 1.04  (0.96-1.12) | 706 | 0.353 | 1.05  (0.97-1.14) | 633 | 1.06  (0.91-1.24) | 172 | 1.06  (0.98-1.14) | 706 | 1.01  (0.88-1.15) | 241 | 1.06  (0.96-1.16) | 465 |
| Breast (postmenopausal) | **1.10**  **(1.07-1.13)** | 4988 | **<.001*** | **1.11**  **(1.08-1.14)** | 4442 | **1.12**  **(1.06-1.19)** | 973 | **1.10**  **(1.07-1.13)** | 4988 | 1.08  (0.99-1.17) | 519 | **1.10**  **(1.07-1.14)** | 4469 |
| Breast (premenopausal) | 1.00  (0.96-1.04) | 2318 | 0.938 | 1.00  (0.95-1.05) | 2097 | 1.02  (0.93-1.12) | 489 | 0.99  (0.95-1.04) | 2318 | 1.00  (0.96-1.05) | 2188 | 0.92  (0.77-1.10) | 130 |
| Cervix | 1.11  (0.97-1.26) | 259 | 0.107^†^ | 0.99  (0.87-1.14) | 210 | **0.67**  **(0.51-0.90)** | 67 | 1.13  (1.00-1.28) | 259 | 1.01  (0.85-1.21) | 127 | 1.04  (0.88-1.23) | 132 |
| Colon | **1.18**  **(1.14-1.23)** | 3368 | **<.001*^,^**^†^ | **1.16**  **(1.12-1.20)** | 3140 | **1.12**  **(1.04-1.20)** | 806 | **1.20**  **(1.15-1.24)** | 3368 | **1.20**  **(1.12-1.29)** | 736 | **1.15**  **(1.10-1.19)** | 2632 |
| Corpus uteri | **1.36**  **(1.30-1.42)** | 1442 | **<.001*** | **1.38**  **(1.32-1.45)** | 1300 | **1.30**  **(1.16-1.46)** | 214 | **1.35**  **(1.29-1.41)** | 1442 | **1.33**  **(1.23-1.44)** | 469 | **1.38**  **(1.31-1.46)** | 973 |
| Esophagus (adeno) | **1.35**  **(1.16-1.56)** | 181 | **0.001*** | **1.37**  **(1.17-1.60)** | 163 | 1.31  (0.99-1.72) | 53 | **1.39**  **(1.20-1.60)** | 181 | **1.45**  **(1.07-1.98)** | 37 | **1.30**  **(1.10-1.53)** | 144 |
| Esophagus (SCC) | **0.71**  **(0.60-0.84)** | 180 | **0.001*** | **0.74**  **(0.62-0.89)** | 155 | **0.65**  **(0.52-0.82)** | 104 | 0.88  (0.70-1.10) | 180 | **0.69**  **(0.49-0.98)** | 46 | **0.70**  **(0.58-0.86)** | 134 |
| Gallbladder | **1.21**  **(1.09-1.35)** | 299 | **0.001*** | **1.21**  **(1.08-1.36)** | 272 | **1.24**  **(1.02-1.52)** | 82 | **1.22**  **(1.10-1.36)** | 299 | 1.22  (0.96-1.55) | 63 | **1.20**  **(1.06-1.35)** | 236 |
| Kidney | **1.25**  **(1.18-1.34)** | 889 | **<.0001*** | **1.29**  **(1.20-1.38)** | 792 | **1.22**  **(1.09-1.37)** | 276 | **1.25**  **(1.18-1.33)** | 889 | **1.27**  **(1.13-1.43)** | 253 | **1.25**  **(1.16-1.35)** | 636 |
| Larynx | 0.91  (0.80-1.04) | 280 | 0.146 | 0.90  (0.78-1.03) | 247 | 0.85  (0.72-1.00) | 192 | 0.90  (0.79-1.03) | 280 | 0.95  (0.74-1.23) | 74 | 0.90  (0.77-1.04) | 206 |
| Lip, Oral cavity, Pharynx | 1.04  (0.94-1.14) | 684 | **0.008**^†^ | 1.06  (0.96-1.16) | 612 | 0.98  (0.83-1.14) | 304 | 1.08  (0.99-1.19) | 684 | 1.06  (0.91-1.23) | 240 | 1.03  (0.91-1.16) | 444 |
| Liver | **1.33**  **(1.21-1.46)** | 392 | **<.001*** | **1.35**  **(1.22-1.48)** | 364 | **1.33**  **(1.14-1.55)** | 139 | **1.36**  **(1.24-1.49)** | 392 | **1.40**  **(1.15-1.70)** | 80 | **1.31**  **(1.18-1.45)** | 312 |
| Lung | 0.98  (0.94-1.02) | 3414 | **<.001*^,^**^†^ | 0.99  (0.95-1.04) | 3174 | 0.98  (0.93-1.04) | 2280 | 1.01  (0.97-1.06) | 3414 | 0.95  (0.87-1.05) | 756 | 0.98  (0.93-1.03) | 2658 |
| Melanoma | 0.99  (0.96-1.03) | 3839 | 0.654 | 0.99  (0.96-1.03) | 3585 | 0.99  (0.92-1.07) | 769 | 0.98  (0.95-1.01) | 3839 | 0.98  (0.92-1.05) | 1040 | 1.00  (0.96-1.04) | 2799 |
| Myeloma | **1.12**  **(1.06-1.19)** | 1597 | **0.001*^,^**^†^ | **1.10**  **(1.04-1.16)** | 1454 | 1.08  (0.97-1.20) | 371 | **1.12**  **(1.06-1.19)** | 1597 | **1.13**  **(1.02-1.25)** | 395 | 1.06  (1.00-1.13) | 1202 |
| Ovary | 1.05  (0.99-1.11) | 1045 | 0.113 | 1.07  (1.00-1.14) | 923 | 1.05  (0.92-1.20) | 200 | 1.05  (0.99-1.12) | 1045 | 1.08  (0.97-1.19) | 368 | 1.03  (0.96-1.11) | 677 |
| Pancreas | **1.12**  **(1.06-1.19)** | 1097 | **<.001*** | **1.13**  **(1.07-1.21)** | 1024 | 1.11  (1.00-1.23) | 362 | **1.11**  **(1.05-1.18)** | 1097 | **1.23**  **(1.09-1.39)** | 239 | **1.09**  **(1.02-1.17)** | 858 |
| Prostate | **0.95**  **(0.93-0.98)** | 6232 | **0.001*** | **0.92**  **(0.89-0.96)** | 5971 | 0.95  (0.90-1.01) | 1575 | **0.95**  **(0.92-0.98)** | 6232 | 0.95  (0.88-1.04) | 239 | **0.93**  **(0.89-0.97)** | 5086 |
| Rectum | **1.08**  **(1.03-1.13)** | 1857 | **0.001*** | **1.09**  **(1.04-1.15)** | 1697 | 1.09  (1.00-1.19) | 514 | **1.09**  **(1.04-1.14)** | 1857 | 1.07  (0.98-1.17) | 488 | **1.08**  **(1.02-1.14)** | 1369 |
| Stomach (cardia) | 1.11  (0.97-1.28) | 215 | 0.126 | 1.16  (1.00-1.33) | 199 | 0.93  (0.75-1.16) | 90 | 1.11  (0.97-1.28) | 215 | 1.20  (0.90-1.58) | 48 | 1.08  (0.92-1.26) | 167 |
| Stomach (non-cardia) | 0.98  (0.87-1.10) | 331 | 0.706 | 1.00  (0.89-1.12) | 306 | 0.89  (0.71-1.10) | 95 | 0.99  (0.89-1.11) | 331 | 1.05  (0.84-1.30) | 87 | 0.95  (0.83-1.09) | 244 |
| Thyroid | 1.05  (0.96-1.15) | 521 | 0.260 | 1.06  (0.97-1.16) | 472 | 0.97  (0.80-1.18) | 106 | 1.08  (0.99-1.18) | 521 | **1.14**  **(1.02-1.28)** | 294 | 0.93  (0.81-1.06) | 227 |
| Total cancer | **1.07**  **(1.05-1.08)** | 47,110 | **<.001*^,^**^†^ | **1.07**  **(1.06-1.08)** | 43,165 | **1.05**  **(1.02-1.07)** | 13,200 | **1.07**  **(1.06-1.09)** | 47,110 | **1.06**  **(1.04-1.08)** | 14,298 | **1.07**  **(1.05-1.08)** | 32,812 |

CI: confidence interval; FU: follow-up time; HR: hazard ratio; n: number of cancer incidence cases; CNS: central nervous system; SCC: squamous cell carcinomas.

^1^ Hazard ratios per 1 SD increment in PC1 from Cox proportional hazards regression using age as the underlying time metric. Stratified by sex; age in 5-year categories and center. Adjusted for education, smoking status and intensity, physical activity, alcohol consumption, and Mediterranean Diet Score. All four principal components were mutually adjusted.

^2^ Crude hazard ratios per 1 SD increment in PC1 from Cox proportional hazards regression using age as the underlying time metric in the complete case dataset (n=340,152). Stratified by sex; age in 5-year categories and center. All four principal components were mutually adjusted.

Bold font indicates statistical significance (p<0.05). * *P*<0.001 indicates Bonferroni-corrected statistical significance (~0.05/96). ^†^ *P* values from spline model because of non-linear relationship.

Supplementary Table 6: Relations of principal component 2 (tall stature; low WHR) per 1 SD increment to cancer incidence in the complete case dataset (n=340,152), after further excluding the initial 2 years of follow-up (n=334,605), in current smokers (n=79,819), fitted as a crude model (n=340,152)^1^, in participants younger than 52.3 years (n=169,473), and older than 52.3 years (n=170,679).

|  | **Cancer incidence** | | | **Cancer incidence** | | **Cancer incidence** | | **Cancer incidence** | | **Cancer incidence** | | **Cancer incidence** | |
| --- | --- | --- | --- | --- | --- | --- | --- | --- | --- | --- | --- | --- | --- |
|  | Complete case dataset | | | FU>2 years | | Current smokers | | Crude model^2^ | | Younger <52.3 years | | Older ≥52.3 years | |
| **Principal component 2** | HR (95% CI) | n | *P* | HR (95% CI) | n | HR (95% CI) | n | HR (95% CI) | n | HR (95% CI) | n | HR (95% CI) | n |
| Bladder | 1.00  (0.95-1.05) | 1414 | 0.967 | 1.00  (0.95-1.06) | 1327 | 0.99  (0.91-1.07) | 608 | 0.96  (0.91-1.01) | 1414 | 0.98  (0.87-1.11) | 279 | 1.01  (0.95-1.07) | 1135 |
| Brain and CNS | 1.08  (1.00-1.16) | 706 | 0.054 | 1.06  (0.98-1.15) | 633 | 1.04  (0.89-1.21) | 172 | 1.06  (0.98-1.14) | 706 | 0.90  (0.79-1.03) | 241 | **1.18**  **(1.07-1.30)** | 465 |
| Breast (postmenopausal) | **1.10**  **(1.07-1.13)** | 4988 | **<.001*** | **1.10**  **(1.07-1.14)** | 4442 | **1.11**  **(1.04-1.18)** | 973 | **1.11**  **(1.08-1.14)** | 4988 | **1.11**  **(1.02-1.22)** | 519 | **1.10**  **(1.07-1.14)** | 4469 |
| Breast (premenopausal) | **1.08**  **(1.03-1.12)** | 2318 | **0.001*** | **1.07**  **(1.02-1.12)** | 2097 | 1.04  (0.95-1.14) | 489 | **1.08**  **(1.04-1.13)** | 2318 | **1.06**  **(1.02-1.11)** | 2188 | **1.25**  **(1.04-1.51)** | 130 |
| Cervix | 1.00  (0.88-1.14) | 259 | 0.945 | 0.98  (0.85-1.13) | 210 | **1.32**  **(1.03-1.70)** | 67 | 0.99  (0.87-1.12) | 259 | 1.15  (0.95-1.38) | 127 | 0.90  (0.75-1.07) | 132 |
| Colon | 1.03  (0.99-1.06) | 3368 | 0.149 | 1.02  (0.99-1.06) | 3140 | 1.02  (0.95-1.09) | 806 | 1.02  (0.99-1.06) | 3368 | 1.06  (0.98-1.14) | 736 | 1.02  (0.98-1.06) | 2632 |
| Corpus uteri | 1.01  (0.96-1.06) | 1442 | 0.776 | 1.02  (0.96-1.08) | 1300 | 0.92  (0.81-1.06) | 214 | 1.02  (0.97-1.08) | 1442 | 1.03  (0.94-1.13) | 469 | 1.00  (0.93-1.06) | 973 |
| Esophagus (adeno) | **0.84**  **(0.73-0.97)** | 181 | **0.016** | 0.86  (0.74-1.00) | 163 | 0.90  (0.69-1.18) | 53 | **0.80**  **(0.70-0.92)** | 181 | 0.95  (0.70-1.29) | 37 | **0.82**  **(0.69-0.96)** | 144 |
| Esophagus (SCC) | 0.95  (0.82-1.11) | 180 | 0.534 | 1.01  (0.86-1.20) | 155 | 0.94  (0.77-1.14) | 104 | 0.87  (0.76-1.01) | 180 | 0.76  (0.57-1.01) | 46 | 1.04  (0.87-1.24) | 134 |
| Gallbladder | 1.08  (0.96-1.22) | 299 | 0.179 | 1.13  (1.00-1.27) | 272 | 0.98  (0.79-1.23) | 82 | 1.06  (0.95-1.19) | 299 | **1.33**  **(1.03-1.71)** | 63 | 1.03  (0.90-1.17) | 236 |
| Kidney | 0.95  (0.89-1.02) | 889 | 0.165 | 0.96  (0.90-1.03) | 792 | 0.90  (0.80-1.02) | 276 | 0.94  (0.89-1.01) | 889 | 0.93  (0.82-1.05) | 253 | 0.97  (0.89-1.05) | 636 |
| Larynx | 0.97  (0.86-1.09) | 280 | 0.580 | **0.78**  **(0.62-0.99)** | 247 | 0.95  (0.83-1.09) | 192 | **0.87**  **(0.78-0.98)** | 280 | 0.92  (0.74-1.15) | 74 | 0.99  (0.86-1.14) | 206 |
| Lip, Oral cavity, Pharynx | **0.86**  **(0.80-0.93)** | 684 | **0.001*** | **0.87**  **(0.80-0.94)** | 612 | **0.69**  **(0.55-0.88)** | 304 | **0.81**  **(0.75-0.87)** | 684 | **0.79**  **(0.69-0.90)** | 240 | **0.90**  **(0.82-0.99)** | 444 |
| Liver | **0.87**  **(0.79-0.96)** | 392 | **0.007** | **0.89**  **(0.80-0.98)** | 364 | 0.87  (0.73-1.03) | 139 | **0.84**  **(0.77-0.93)** | 392 | **0.80**  **(0.64-0.99)** | 80 | 0.89  (0.80-1.00) | 312 |
| Lung | 0.97  (0.94-1.00) | 3414 | 0.070 | 0.97  (0.94-1.01) | 3174 | **0.95**  **(0.91-0.99)** | 2280 | **0.87**  **(0.84-0.90)** | 3414 | 1.01  (0.94-1.08) | 756 | 0.96  (0.92-1.00) | 2658 |
| Melanoma | **1.09**  **(1.05-1.13)** | 3839 | **<.001*** | **1.14**  **(1.09-1.19)** | 3585 | **1.16**  **(1.07-1.24)** | 769 | **1.11**  **(1.07-1.14)** | 3839 | **1.15**  **(1.08-1.23)** | 1040 | **1.07**  **(1.03-1.11)** | 2799 |
| Myeloma | 1.05  (1.00-1.10) | 1597 | 0.076 | 1.04  (0.98-1.09) | 1454 | 1.08  (0.97-1.20) | 371 | 1.05  (0.99-1.10) | 1597 | 1.07  (0.97-1.19) | 395 | 1.04  (0.98-1.10) | 1202 |
| Ovary | 1.05  (0.99-1.12) | 1045 | 0.123 | 1.04  (0.97-1.12) | 923 | 1.03  (0.89-1.19) | 200 | 1.05  (0.99-1.12) | 1045 | 1.04  (0.93-1.16) | 368 | 1.06  (0.98-1.15) | 677 |
| Pancreas | 1.00  (0.94-1.06) | 1097 | 0.981 | 0.99  (0.93-1.05) | 1024 | 0.94  (0.85-1.05) | 362 | 0.98  (0.93-1.04) | 1097 | 1.06  (0.93-1.20) | 239 | 0.99  (0.92-1.06) | 858 |
| Prostate | 1.01  (0.98-1.03) | 6232 | 0.601 | 1.01  (0.98-1.04) | 5971 | 1.00  (0.95-1.05) | 1575 | 1.02  (0.99-1.05) | 6232 | 0.99  (0.93-1.05) | 239 | 1.01  (0.99-1.04) | 5086 |
| Rectum | **0.90**  **(0.86-0.94)** | 1857 | **<.001*** | **0.90**  **(0.86-0.95)** | 1697 | 0.99  (0.90-1.08) | 514 | **0.89**  **(0.85-0.94)** | 1857 | 0.94  (0.86-1.03) | 488 | **0.88**  **(0.84-0.93)** | 1369 |
| Stomach (cardia) | 0.98  (0.85-1.12) | 215 | 0.721 | 0.95  (0.83-1.09) | 199 | 0.93  (0.75-1.14) | 90 | 0.94  (0.82-1.07) | 215 | 0.82  (0.62-1.07) | 48 | 1.04  (0.89-1.21) | 167 |
| Stomach (non-cardia) | **0.88**  **(0.79-0.98)** | 331 | **0.022** | **0.85**  **(0.76-0.95)** | 306 | 0.97  (0.78-1.19) | 95 | **0.84**  **(0.75-0.93)** | 331 | 0.82  (0.66-1.02) | 87 | 0.90  (0.79-1.02) | 244 |
| Thyroid | **1.14**  **(1.05-1.25)** | 521 | **0.003** | **1.13**  **(1.03-1.24)** | 472 | 1.19  (0.98-1.46) | 106 | **1.12**  **(1.03-1.22)** | 521 | 1.07  (0.96-1.21) | 294 | **1.24**  **(1.09-1.43)** | 227 |
| Total cancer | **1.03**  **(1.02-1.04)** | 47,110 | **<.001*** | **1.03**  **(1.02-1.04)** | 43,165 | 1.00  (0.99-1.02) | 13,200 | **1.01**  **(1.01-1.02)** | 47,110 | **1.03**  **(1.02-1.05)** | 14,298 | **1.02**  **(1.01-1.03)** | 32,812 |

CI: confidence interval; FU: follow-up time; HR: hazard ratio; n: number of cancer incidence cases; CNS: central nervous system; SCC: squamous cell carcinomas.

^1^ Hazard ratios per 1 SD increment in PC2 from Cox proportional hazards regression using age as the underlying time metric. Stratified by sex; age in 5-year categories and center. Adjusted for education, smoking status and intensity, physical activity, alcohol consumption, and Mediterranean Diet Score. All four principal components were mutually adjusted.

^2^ Crude hazard ratios per 1 SD increment in PC2 from Cox proportional hazards regression using age as the underlying time metric in the complete case dataset (n=340,152). Stratified by sex; age in 5-year categories and center. All four principal components were mutually adjusted.

Bold font indicates statistical significance (p<0.05); * *P*<0.001 indicates Bonferroni-corrected statistical significance (~0.05/96).

Supplementary Table 7: Relations of principal component 3 (tall stature; high WHR) per 1 SD increment to cancer incidence in the complete case dataset (n=340,152), after further excluding the initial 2 years of follow-up (n=334,605), in current smokers (n=79,819), fitted as a crude model (n=340,152)^1^, in participants younger than 52.3 years (n=169,473), and older than 52.3 years (n=170,679).

|  | **Cancer incidence** | | | **Cancer incidence** | | **Cancer incidence** | | **Cancer incidence** | | **Cancer incidence** | | **Cancer incidence** | |
| --- | --- | --- | --- | --- | --- | --- | --- | --- | --- | --- | --- | --- | --- |
|  | Complete case dataset | | | FU>2 years | | Current smokers | | Crude model^2^ | | Younger <52.3 years | | Older ≥52.3 years | |
| **Principal component 3** | HR (95% CI) | n | *P* | HR (95% CI) | n | HR (95% CI) | n | HR (95% CI) | n | HR (95% CI) | n | HR (95% CI) | n |
| Bladder | 1.03  (0.97-1.09) | 1414 | 0.398 | 1.02  (0.96-1.08) | 1327 | 0.99  (0.91-1.09) | 608 | **1.09**  **(1.03-1.15)** | 1414 | 1.03  (0.90-1.18) | 279 | 1.02  (0.96-1.09) | 1135 |
| Brain and CNS | 1.00  (0.92-1.08) | 706 | 0.990 | 1.00  (0.92-1.09) | 633 | 1.06  (0.91-1.25) | 172 | 1.00  (0.92-1.08) | 706 | 1.01  (0.88-1.16) | 241 | 1.00  (0.91-1.10) | 465 |
| Breast (postmenopausal) | 1.00  (0.97-1.03) | 4988 | 0.886 | 1.00  (0.97-1.03) | 4442 | 0.96  (0.91-1.03) | 973 | 1.01  (0.98-1.04) | 4988 | 0.97  (0.89-1.06) | 519 | 1.00  (0.97-1.03) | 4469 |
| Breast (premenopausal) | 1.03  (0.98-1.07) | 2318 | 0.261 | 1.02  (0.97-1.06) | 2097 | 1.03  (0.94-1.13) | 489 | 1.04  (1.00-1.08) | 2318 | 1.03  (0.98-1.07) | 2188 | 1.02  (0.85-1.22) | 130 |
| Cervix | 1.00  (0.89-1.13) | 259 | 0.992 | 0.97  (0.84-1.10) | 210 | 1.23  (0.95-1.58) | 67 | 1.01  (0.90-1.14) | 259 | 0.94  (0.79-1.13) | 127 | 1.04  (0.88-1.22) | 132 |
| Colon | **1.09**  **(1.05-1.13)** | 3368 | **<.001*** | **1.08**  **(1.04-1.12)** | 3140 | 1.06  (0.99-1.15) | 806 | **1.10**  **(1.07-1.14)** | 3368 | 1.04  (0.96-1.12) | 736 | **1.10**  **(1.06-1.15)** | 2632 |
| Corpus uteri | **0.92**  **(0.87-0.97)** | 1442 | **<.001*** | **0.92**  **(0.87-0.97)** | 1300 | 0.88  (0.77-1.00) | 214 | **0.91**  **(0.87-0.96)** | 1442 | 0.93  (0.85-1.02) | 469 | **0.91**  **(0.86-0.97)** | 973 |
| Esophagus (adeno) | **1.32**  **(1.14-1.54)** | 181 | **<.001*** | **1.35**  **(1.15-1.58)** | 163 | 1.35  (1.00-1.81) | 53 | **1.34**  **(1.15-1.55)** | 181 | **1.42**  **(1.02-1.96)** | 37 | **1.29**  **(1.09-1.52)** | 144 |
| Esophagus (SCC) | **1.32**  **(1.12-1.55)** | 180 | **0.001*** | **1.31**  **(1.10-1.56)** | 155 | **1.53**  **(1.23-1.90)** | 104 | **1.52**  **(1.30-1.77)** | 180 | **1.85**  **(1.36-2.52)** | 46 | 1.18  (0.98-1.42) | 134 |
| Gallbladder | 1.05  (0.93-1.18) | 299 | 0.420 | 1.06  (0.94-1.20) | 272 | 0.97  (0.77-1.21) | 82 | 1.06  (0.95-1.19) | 299 | 1.07  (0.82-1.40) | 63 | 1.04  (0.92-1.19) | 236 |
| Kidney | **1.11**  **(1.04-1.19)** | 889 | **0.002** | **1.09**  **(1.01-1.17)** | 792 | 1.11  (0.98-1.27) | 276 | **1.13**  **(1.05-1.21)** | 889 | **1.15**  **(1.01-1.31)** | 253 | **1.09**  **(1.01-1.19)** | 636 |
| Larynx | **1.33**  **(1.16-1.51)** | 280 | **<.001*** | **1.35**  **(1.17-1.55)** | 247 | **1.43**  **(1.22-1.67)** | 192 | **1.54**  **(1.36-1.75)** | 280 | **1.62**  **(1.26-2.09)** | 74 | **1.23**  **(1.06-1.44)** | 206 |
| Lip, Oral cavity, Pharynx | **1.16**  **(1.07-1.26)** | 684 | **<.001*** | **1.12**  **(1.02-1.22)** | 612 | **1.42**  **(1.25-1.61)** | 304 | **1.25**  **(1.15-1.35)** | 684 | 1.11  (0.96-1.28) | 240 | **1.17**  **(1.06-1.30)** | 444 |
| Liver | 1.02  (0.92-1.13) | 392 | 0.694 | 1.03  (0.93-1.14) | 364 | 1.02  (0.86-1.21) | 139 | 1.07  (0.97-1.18) | 392 | 0.98  (0.79-1.23) | 80 | 1.02  (0.91-1.15) | 312 |
| Lung | **1.14**  **(1.10-1.18)** | 3414 | **<.001*** | **1.12**  **(1.08-1.17)** | 3174 | **1.19**  **(1.14-1.24)** | 2280 | **1.29**  **(1.25-1.34)** | 3414 | **1.16**  **(1.07-1.25)** | 756 | **1.13**  **(1.08-1.18)** | 2658 |
| Melanoma | **1.05**  **(1.02-1.09)** | 3839 | **0.003** | **1.06**  **(1.02-1.10)** | 3585 | 1.02  (0.94-1.10) | 769 | **1.05**  **(1.02-1.09)** | 3839 | **1.08**  **(1.01-1.15)** | 1040 | 1.04  (1.00-1.08) | 2799 |
| Myeloma | 1.03  (0.98-1.08) | 1597 | 0.289 | 1.02  (0.97-1.08) | 1454 | 1.03  (0.92-1.16) | 371 | 1.03  (0.98-1.08) | 1597 | 0.99  (0.89-1.10) | 395 | 1.04  (0.98-1.10) | 1202 |
| Ovary | 0.99  (0.93-1.05) | 1045 | 0.641 | 0.98  (0.92-1.05) | 923 | 0.99  (0.86-1.13) | 200 | 0.98  (0.93-1.05) | 1045 | 1.07  (0.96-1.19) | 368 | 0.95  (0.88-1.02) | 677 |
| Pancreas | **1.10**  **(1.03-1.17)** | 1097 | **0.002** | **1.10**  **(1.03-1.17)** | 1024 | 1.07  (0.96-1.20) | 362 | **1.13**  **(1.06-1.20)** | 1097 | 1.09  (0.95-1.25) | 239 | **1.11**  **(1.03-1.19)** | 858 |
| Prostate | **1.04**  **(1.01-1.08)** | 6232 | **0.005** | **1.04**  **(1.01-1.07)** | 5971 | 1.05  (0.99-1.12) | 1575 | **1.04**  **(1.01-1.07)** | 6232 | 1.05  (0.97-1.13) | 239 | 1.04  (1.00-1.07) | 5086 |
| Rectum | 1.04  (0.99-1.09) | 1857 | 0.156 | 1.04  (0.99-1.09) | 1697 | 1.02  (0.92-1.12) | 514 | 1.05  (1.00-1.10) | 1857 | 1.00  (0.91-1.11) | 488 | 1.05  (0.99-1.11) | 1369 |
| Stomach (cardia) | **1.19**  **(1.03-1.37)** | 215 | **0.020** | 1.17  (1.00-1.35) | 199 | **1.29**  **(1.03-1.61)** | 90 | **1.25**  **(1.08-1.44)** | 215 | **1.49**  **(1.11-1.99)** | 48 | 1.11  (0.94-1.31) | 167 |
| Stomach (non-cardia) | 0.97  (0.86-1.08) | 331 | 0.549 | 0.93  (0.83-1.05) | 306 | 0.91  (0.72-1.15) | 95 | 0.97  (0.86-1.08) | 331 | 1.02  (0.81-1.27) | 87 | 0.96  (0.84-1.09) | 244 |
| Thyroid | **1.17**  **(1.07-1.27)** | 521 | **0.001*** | 1.15  (1.05-1.26) | 472 | 1.09  (0.89-1.33) | 106 | **1.15**  **(1.05-1.25)** | 521 | **1.23**  **(1.10-1.37)** | 294 | 1.10  (0.96-1.26) | 227 |
| Total cancer | **1.04**  **(1.03-1.05)** | 47,110 | **<.001*** | **1.04**  **(1.03-1.05)** | 43,165 | **1.07**  **(1.05-1.09)** | 13,200 | **1.06**  **(1.05-1.07)** | 47,110 | **1.05**  **(1.03-1.07)** | 14,298 | **1.04**  **(1.03-1.05)** | 32,812 |

CI: confidence interval; FU: follow-up time; HR: hazard ratio; n: number of cancer incidence cases; CNS: central nervous system; SCC: squamous cell carcinomas.

^1^ Hazard ratios per 1 SD increment in PC3 from Cox proportional hazards regression using age as the underlying time metric. Stratified by sex; age in 5-year categories and center. Adjusted for education, smoking status and intensity, physical activity, alcohol consumption, and Mediterranean Diet Score. All four principal components were mutually adjusted.

^2^ Crude hazard ratios per 1 SD increment in PC3 from Cox proportional hazards regression using age as the underlying time metric in the complete case dataset (n=340,152). Stratified by sex; age in 5-year categories and center. All four principal components were mutually adjusted.

Bold font indicates statistical significance (p<0.05); * *P*<0.001 indicates Bonferroni-corrected statistical significance (~0.05/96).

Supplementary Table 8: Relations of principal component 4 (high BMI and weight; low WC and HC) per 1 SD increment to cancer incidence in the complete case dataset (n=340,152), after further excluding the initial 2 years of follow-up (n=334,605), in current smokers (n=79,819), fitted as a crude model (n=340,152)^1^, in participants younger than 52.3 years (n=169,473), and older than 52.3 years (n=170,679).

|  | **Cancer incidence** | | | **Cancer incidence** | | **Cancer incidence** | | **Cancer incidence** | | **Cancer incidence** | | **Cancer incidence** | |
| --- | --- | --- | --- | --- | --- | --- | --- | --- | --- | --- | --- | --- | --- |
|  | Complete case dataset | | | FU>2 years | | Current smokers | | Crude model^2^ | | Younger <52.3 years | | Older ≥52.3 years | |
| **Principal component 4** | HR (95% CI) | n | *P* | HR (95% CI) | n | HR (95% CI) | n | HR (95% CI) | n | HR (95% CI) | n | HR (95% CI) | n |
| Bladder | 1.01  (0.96-1.07) | 1414 | 0.697 | 1.02  (0.96-1.08) | 1327 | 1.02  (0.93-1.11) | 608 | 1.00  (0.94-1.05) | 1414 | 1.01  (0.89-1.14) | 279 | 1.01  (0.95-1.07) | 1135 |
| Brain and CNS | 0.96  (0.89-1.04) | 706 | 0.312 | 0.97  (0.89-1.05) | 633 | 1.01  (0.85-1.20) | 172 | 0.96  (0.88-1.04) | 706 | 0.92  (0.81-1.05) | 241 | 0.98  (0.89-1.09) | 465 |
| Breast (postmenopausal) | 1.03  (1.00-1.06) | 4988 | 0.085 | 1.04  (1.00-1.07) | 4442 | 1.01  (0.94-1.08) | 973 | 1.02  (0.99-1.05) | 4988 | 1.01  (0.92-1.11) | 519 | 1.03  (1.00-1.06) | 4469 |
| Breast (premenopausal) | 0.98  (0.93-1.02) | 2318 | 0.287 | 0.98  (0.94-1.03) | 2097 | 1.03  (0.93-1.14) | 489 | 0.98  (0.93-1.02) | 2318 | 0.97  (0.93-1.02) | 2188 | 1.01  (0.82-1.24) | 130 |
| Cervix | 1.06  (0.94-1.21) | 259 | 0.330 | 1.13  (0.99-1.30) | 210 | 0.94  (0.69-1.27) | 67 | 1.06  (0.94-1.20) | 259 | 1.07  (0.90-1.28) | 127 | 1.05  (0.87-1.26) | 132 |
| Colon | 0.99  (0.95-1.03) | 3368 | 0.562 | 0.98  (0.95-1.02) | 3140 | 0.98  (0.91-1.06) | 806 | 0.98  (0.95-1.02) | 3368 | 1.02  (0.94-1.10) | 736 | 0.98  (0.94-1.02) | 2632 |
| Corpus uteri | 1.06  (1.00-1.11) | 1442 | **0.033** | **1.06**  **(1.01-1.12)** | 1300 | 1.09  (0.94-1.26) | 214 | 1.06  (1.00-1.11) | 1442 | 1.06  (0.97-1.16) | 469 | 1.06  (1.00-1.13) | 973 |
| Esophagus (adeno) | 0.94  (0.81-1.09) | 181 | 0.395 | 0.92  (0.79-1.07) | 163 | 0.80  (0.60-1.08) | 53 | 0.93  (0.80-1.08) | 181 | 1.03  (0.77-1.38) | 37 | 0.90  (0.76-1.06) | 144 |
| Esophagus (SCC) | 0.88  (0.75-1.03) | 180 | 0.110 | 0.89  (0.75-1.06) | 155 | **0.76**  **(0.61-0.95)** | 104 | 0.87  (0.74-1.02) | 180 | 0.94  (0.70-1.25) | 46 | 0.85  (0.70-1.03) | 134 |
| Gallbladder | 0.98  (0.87-1.10) | 299 | 0.712 | 0.96  (0.85-1.10) | 272 | 1.01  (0.79-1.27) | 82 | 0.97  (0.86-1.10) | 299 | 0.99  (0.76-1.28) | 63 | 0.97  (0.85-1.11) | 236 |
| Kidney | 1.07  (1.00-1.15) | 889 | 0.053 | 1.07  (1.00-1.16) | 792 | 1.04  (0.91-1.18) | 276 | 1.06  (0.99-1.14) | 889 | 1.03  (0.91-1.17) | 253 | **1.10**  **(1.01-1.19)** | 636 |
| Larynx | 0.94  (0.83-1.07) | 280 | 0.350 | 0.97  (0.85-1.11) | 247 | 0.97  (0.83-1.14) | 192 | 0.90  (0.79-1.03) | 280 | 0.94  (0.74-1.19) | 74 | 0.93  (0.80-1.09) | 206 |
| Lip, Oral cavity, Pharynx | 0.93  (0.86-1.01) | 684 | 0.081 | 0.94  (0.86-1.02) | 612 | 0.88  (0.73-1.06) | 304 | **0.92**  **(0.85-0.99)** | 684 | 0.96  (0.83-1.09) | 240 | 0.92  (0.83-1.02) | 444 |
| Liver | 0.94  (0.85-1.04) | 392 | 0.238 | 0.96  (0.86-1.06) | 364 | 0.93  (0.78-1.10) | 139 | 0.93  (0.84-1.03) | 392 | **1.27**  **(1.03-1.58)** | 80 | **0.87**  **(0.77-0.97)** | 312 |
| Lung | **0.89**  **(0.85-0.92)** | 3414 | **<.001*** | **0.89**  **(0.85-0.92)** | 3174 | **0.87**  **(0.83-0.92)** | 2280 | **0.85**  **(0.82-0.88)** | 3414 | 0.98  (0.90-1.06) | 756 | **0.86**  **(0.82-0.89)** | 2658 |
| Melanoma | **1.05**  **(1.01-1.08)** | 3839 | **0.006** | **1.04**  **(1.01-1.08)** | 3585 | 1.06  (0.97-1.14) | 769 | **1.05**  **(1.02-1.09)** | 3839 | **1.08**  **(1.02-1.15)** | 1040 | 1.04  (1.00-1.08) | 2799 |
| Myeloma | 0.99  (0.94-1.05) | 1597 | 0.794 | 0.99  (0.94-1.05) | 1454 | 0.97  (0.87-1.09) | 371 | 0.99  (0.94-1.05) | 1597 | 0.96  (0.87-1.07) | 395 | 1.00  (0.94-1.06) | 1202 |
| Ovary | **1.11**  **(1.03-1.20)** | 1045 | **0.007**^†^ | **1.10**  **(1.01-1.19)** | 923 | 1.12  (0.96-1.31) | 200 | **1.11**  **(1.03-1.20)** | 1045 | 1.10  (0.99-1.22) | 368 | 0.99  (0.91-1.07) | 677 |
| Pancreas | 0.99  (0.93-1.05) | 1097 | 0.702 | 0.99  (0.93-1.06) | 1024 | 1.01  (0.90-1.14) | 362 | 0.98  (0.92-1.05) | 1097 | 0.92  (0.81-1.06) | 239 | 1.01  (0.94-1.08) | 858 |
| Prostate | 1.02  (0.99-1.05) | 6232 | 0.229 | 1.03  (1.00-1.06) | 5971 | 1.03  (0.97-1.09) | 1575 | 1.02  (0.99-1.05) | 6232 | 1.03  (0.96-1.10) | 239 | 1.02  (0.99-1.05) | 5086 |
| Rectum | 0.98  (0.94-1.03) | 1857 | 0.506 | **0.92**  **(0.85-0.99)** | 1697 | 0.99  (0.90-1.09) | 514 | 0.98  (0.94-1.03) | 1857 | 0.98  (0.89-1.08) | 488 | 0.98  (0.92-1.04) | 1369 |
| Stomach (cardia) | 1.04  (0.90-1.20) | 215 | 0.592 | 1.03  (0.89-1.20) | 199 | 1.15  (0.91-1.45) | 90 | 1.04  (0.90-1.20) | 215 | 1.19  (0.88-1.60) | 48 | 0.99  (0.84-1.18) | 167 |
| Stomach (non-cardia) | 1.08  (0.96-1.21) | 331 | 0.222 | 1.09  (0.97-1.24) | 306 | 1.02  (0.81-1.29) | 95 | 1.07  (0.95-1.21) | 331 | 1.10  (0.88-1.38) | 87 | 1.06  (0.92-1.22) | 244 |
| Thyroid | 1.10  (1.00-1.21) | 521 | **0.048** | 1.12  (1.01-1.23) | 472 | 1.08  (0.87-1.35) | 106 | 1.09  (1.00-1.20) | 521 | 1.06  (0.94-1.20) | 294 | 1.14  (0.99-1.33) | 227 |
| Total cancer | 1.00  (0.99-1.01) | 47,110 | 0.890 | 1.00  (0.99-1.01) | 43,165 | 0.98  (0.96-1.00) | 13,200 | 0.99  (0.99-1.00) | 47,110 | 1.01  (0.99-1.03) | 14,298 | 0.99  (0.98-1.01) | 32,812 |

CI: confidence interval; FU: follow-up time; HR: hazard ratio; n: number of cancer incidence cases; CNS: central nervous system; SCC: squamous cell carcinomas.

^1^ Hazard ratios per 1 SD increment in PC4 from Cox proportional hazards regression using age as the underlying time metric. Stratified by sex; age in 5-year categories and center. Adjusted for education, smoking status and intensity, physical activity, alcohol consumption, and Mediterranean Diet Score. All four principal components were mutually adjusted.

^2^ Crude hazard ratios per 1 SD increment in PC4 from Cox proportional hazards regression using age as the underlying time metric in the complete case dataset (n=340,152). Stratified by sex; age in 5-year categories and center. All four principal components were mutually adjusted.

Bold font indicates statistical significance (p<0.05); ^*^ *P* 0.001 indicates Bonferroni-corrected statistical significance (~0.05/96). ^†^ *P* values from spline model because of non-linear relationship.

**
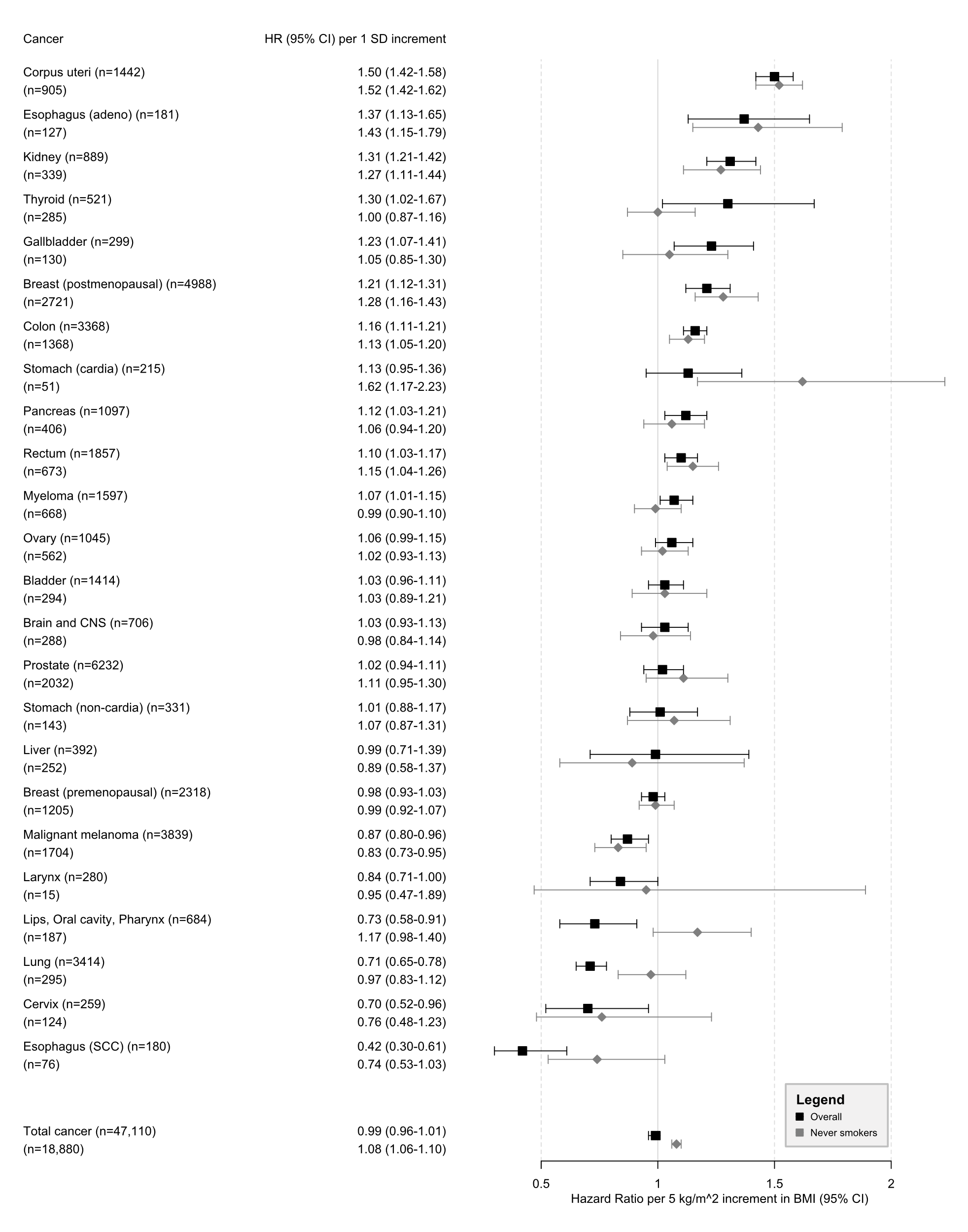
**

Supplementary Figure 9: Hazard ratios (HRs) for total cancer and 24 cancer subtypes per 5 kg/m^2^ increment in body mass index (BMI) with corresponding 95% confidence intervals (95% CIs) from Cox proportional hazards regressions in the total population (n=340,152) and in never smokers (n=160,111); n: number of cancer incidence cases; CNS: central nervous system; SCC: squamous cell carcinomas.

Supplementary Table 9: Median anthropometric measures and median values for each principal component (PC) for participants who never smoked (n=160,111) and current smokers (n=79,819).

|  | Never smoking participants | Smoking participants |
| --- | --- | --- |
| **Men** | (n=37,139) | (n=35,272) |
| Height [cm] | 175.0 | 174.5 |
| Weight [kg] | 78.7 | 79.1 |
| Body mass index [kg/m^2^] | 25.7 | 26.0 |
| Waist circumference [cm] | 92.0 | 94.0 |
| Hip circumference [cm] | 99.9 | 99.5 |
| Waist-to-hip ratio | 0.92 | 0.94 |
| PC1 | -0.122 | -0.117 |
| PC2 | 0.066 | -0.073 |
| PC3 | -0.117 | 0.055 |
| PC4 | -0.056 | -0.011 |
| **Women** | (n=122,972) | (n=44,547) |
| Height [cm] | 161.4 | 162.5 |
| Weight [kg] | 64.5 | 63.8 |
| Body mass index [kg/m^2^] | 24.7 | 24.1 |
| Waist circumference [cm] | 78.0 | 77.5 |
| Hip circumference [cm] | 100.0 | 98.1 |
| Waist-to-hip ratio | 0.78 | 0.79 |
| PC1 | -0.131 | -0.270 |
| PC2 | 0.003 | -0.039 |
| PC3 | -0.058 | 0.171 |
| PC4 | -0.017 | -0.006 |

Literature Cited

1. Riboli E, Hunt KJ, Slimani N, Ferrari P, Norat T, Fahey M et al. European Prospective Investigation into Cancer and Nutrition (EPIC): study populations and data collection. Public Health Nutr 2002; 5(6B):1113–24.

2. Wareham NJ, Jakes RW, Rennie KL, Schuit J, Mitchell J, Hennings S et al. Validity and repeatability of a simple index derived from the short physical activity questionnaire used in the European Prospective Investigation into Cancer and Nutrition (EPIC) study. Public Health Nutr 2003; 6(4):407–13.

3. Couto E, Boffetta P, Lagiou P, Ferrari P, Buckland G, Overvad K et al. Mediterranean dietary pattern and cancer risk in the EPIC cohort. Br J Cancer 2011; 104(9):1493–9.

4. Hernán MA, Hernández-Díaz S, Robins JM. A Structural Approach to Selection Bias. Epidemiology 2004; 15(5):615–25.

5. Greenland S, Pearl J, Robins JM. Causal diagrams for epidemiologic research. Epidemiology 1999; 10(1):37–48.

6. Textor J, van der Zander B, Gilthorpe MS, Liskiewicz M, Ellison GT. Robust causal inference using directed acyclic graphs: the R package 'dagitty'. Int J Epidemiol 2016; 45(6):1887–94.
